# Counterbalancing anti-adhesive effects of Tenascin-C through fibronectin expression in endothelial cells

**DOI:** 10.1038/s41598-017-13008-9

**Published:** 2017-10-06

**Authors:** Agata Radwanska, Dominique Grall, Sébastien Schaub, Stéphanie Beghelli-de la Forest Divonne, Delphine Ciais, Samah Rekima, Tristan Rupp, Anne Sudaka, Gertraud Orend, Ellen Van Obberghen-Schilling

**Affiliations:** 1 Université Côte d’Azur, CNRS, INSERM, iBV, France; 2 Centre Antoine Lacassagne, Nice, 06189 France; 3 Inserm, U1109, MN3T laboratory, The Microenvironmental Niche in Tumorigenesis and Targeted Therapy, Strasbourg, F-67000 France; 4 University Strasbourg, Strasbourg, F-67000 France

## Abstract

Cellular fibronectin (FN) and tenascin-C (TNC) are prominent development- and disease-associated matrix components with pro- and anti-adhesive activity, respectively. Whereas both are present in the tumour vasculature, their functional interplay on vascular endothelial cells remains unclear. We have previously shown that basally-oriented deposition of a FN matrix restricts motility and promotes junctional stability in cultured endothelial cells and that this effect is tightly coupled to expression of FN. Here we report that TNC induces FN expression in endothelial cells. This effect counteracts the potent anti-adhesive activity of TNC and leads to the assembly of a dense highly-branched subendothelial matrix that enhances tubulogenic activity. These findings suggest that pro-angiogenic remodelling of the perivascular matrix may involve TNC-induced upregulation of FN in endothelial cells.

## Introduction

Angiogenesis, the sprouting of new vasculature from a pre-existing vascular network, is an essential process during development, maintenance of tissues and metastatic spread of cancer. This multi-step process is tightly regulated and spatiotemporally controlled by various soluble cytokines, membrane-bound proteins, cell-matrix and cell-cell interactions and hemodynamic forces. In recent years it has become clear that dynamic remodelling of the extracellular matrix (ECM) is essential for all stages of angiogenesis. Through adhesive interactions with integrins expressed on the endothelial cell surface, the ECM orchestrates complex signalling cascades within the cells and affects many fundamental aspects of their biology, including proliferation, migration, cytoskeletal organization, cell shape, survival, and ultimately blood vessel stabilization (reviewed in^1^). Tenascin-C (TNC) and alternatively spliced forms of fibronectin (FN) are principle ECM components of the angiogenic vasculature of tumours, yet barely detected in quiescent adult vessels (reviewed in^2^). Genetic studies in mice and fish have pointed to a fundamental role for FN and its primary receptor α5β1 integrin in early blood vessel development and vascular physio-pathology (reviewed in^3,4^). FN-null mice die at embryonic day 9.5 with severe cardiovascular defects^5^ and α5 null mice display the most severe vascular defects of all the null phenotypes of α-encoding integrin genes^6^. Although TNC knockout mice do not display an embryonic lethal phenotype^7,8^, TNC expression is highly associated with angiogenesis in a wide range of disease states, including cancer^9–11^.

Adhesive and counter-adhesive effects are attributed respectively to FN and TNC. One mechanism by which TNC modulates cell adhesion-dependent processes involves its direct interaction with FN, which leads to interference of FN binding to syndecan-4^12^. TNC can also interact with cognate integrins on the surface of cells^13^ (and references therein). Endothelial cells express TNC-binding integrin αvβ3^3^. αvβ3 is upregulated in tumour-associated blood vessels where it has been found to play both pro- and anti-angiogenic roles in tumour angiogenesis, depending on the context^14^.

FN matrix assembly, or fibrillogenesis, is a complex process (reviewed in^15,16^) driven by α5β1 integrin that takes place at specialized integrin-based structures called fibrillar adhesions at the cell-matrix interface^17–19^. In the context of blood vessel remodelling, FN deposited by endothelial cells forms a pericellular network of fibrils that provides a mechanically ideal support for promoting neovessel development^20^. Moreover, the FN scaffold can modulate angiogenic signalling by sequestering and increasing the bioavailability of diffused factors, as it binds most of the growth factors from the platelet-derived growth factor, vascular endothelial growth factor (VEGF) and fibroblast growth factor families^21–23^. Cellular FN variants are expressed around tumour blood vessels^24–26^ and we have previously shown that FN assembly by endothelial cells is a cell-autonomous process coupled to expression of the protein^27^. Here we show that vascular endothelial cells respond to a direct anti-adhesive effect of TNC by enhancing FN expression and assembly.

## Results

### Different localization of FN and TNC in angiogenic blood vessels of human tumours

**Figure 1:**
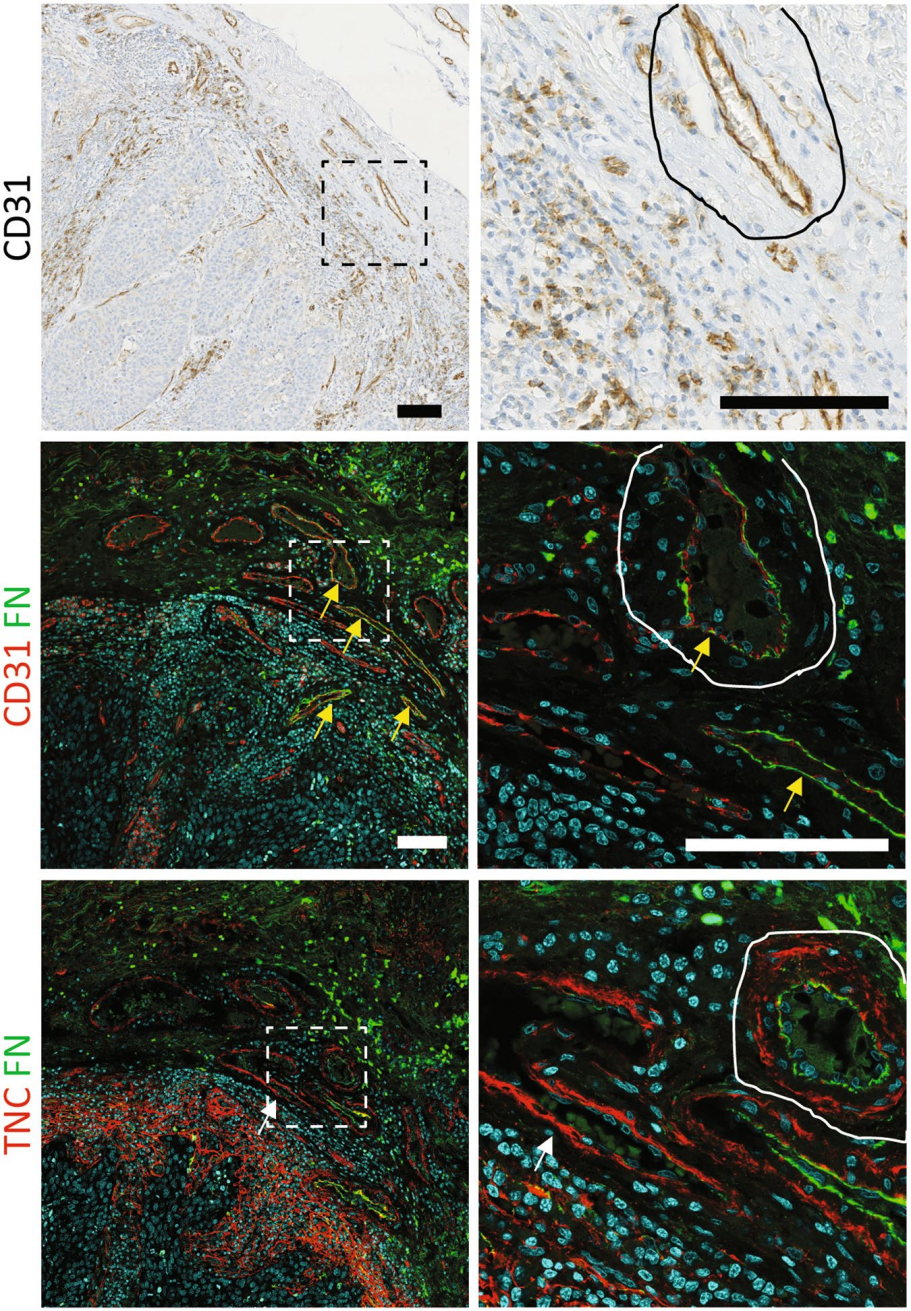
FN and TNC are expressed in angiogenic blood vessels of human tumours. (top) CD31 immunohistochemical staining (brown) of human HNSCC counterstained with haematoxylin (blue). Double immunofluorescent staining, as indicated, of FN with CD31 or TNC on adjacent sections of the same tumour are shown. Separate images for the FN/CD31 and FN/TNC channels, are shown as Supplementary Information (Fig. S1). Nuclei are stained with DRAQ5 (blue). FN-expressing vessels (yellow arrows) and TNC-positive/FN-negative vessels (white arrows) are indicated. Dotted squares (left images) depict zoomed areas (right images). Corresponding areas of the same vessel are encircled. Bars = 100 µm.

To determine the expression and relative localization of FN and TNC in the vasculature of human tumours, we performed immunostaining (Fig. 1 and Supplementary Fig. S1) on adjacent sections of head and neck squamous cell carcinomas (HNSCC). Double immunofluorescence staining of FN and CD31 confirmed the association of FN with a subset of tumour-associated microvessels (yellow arrows). TNC was present around the same vessels (TNC-FN co-staining). Whereas FN directly ensheathed the endothelial cells, TNC was localized on the abluminal side of the vascular basement membrane. These results are consistent with previous observations^24^ and suggest that TNC is derived from perivascular cells. However, some vessels displayed little or no FN staining and TNC appeared to be in direct contact with cells lining the vessels (Fig. 1, white arrow). Together these observations reflect the heterogeneity of the tumour vasculature and raise questions concerning the dynamic regulation of matrix protein expression by vascular endothelial cells.

### FN and TNC expression in endothelial cells

**Figure 2:**
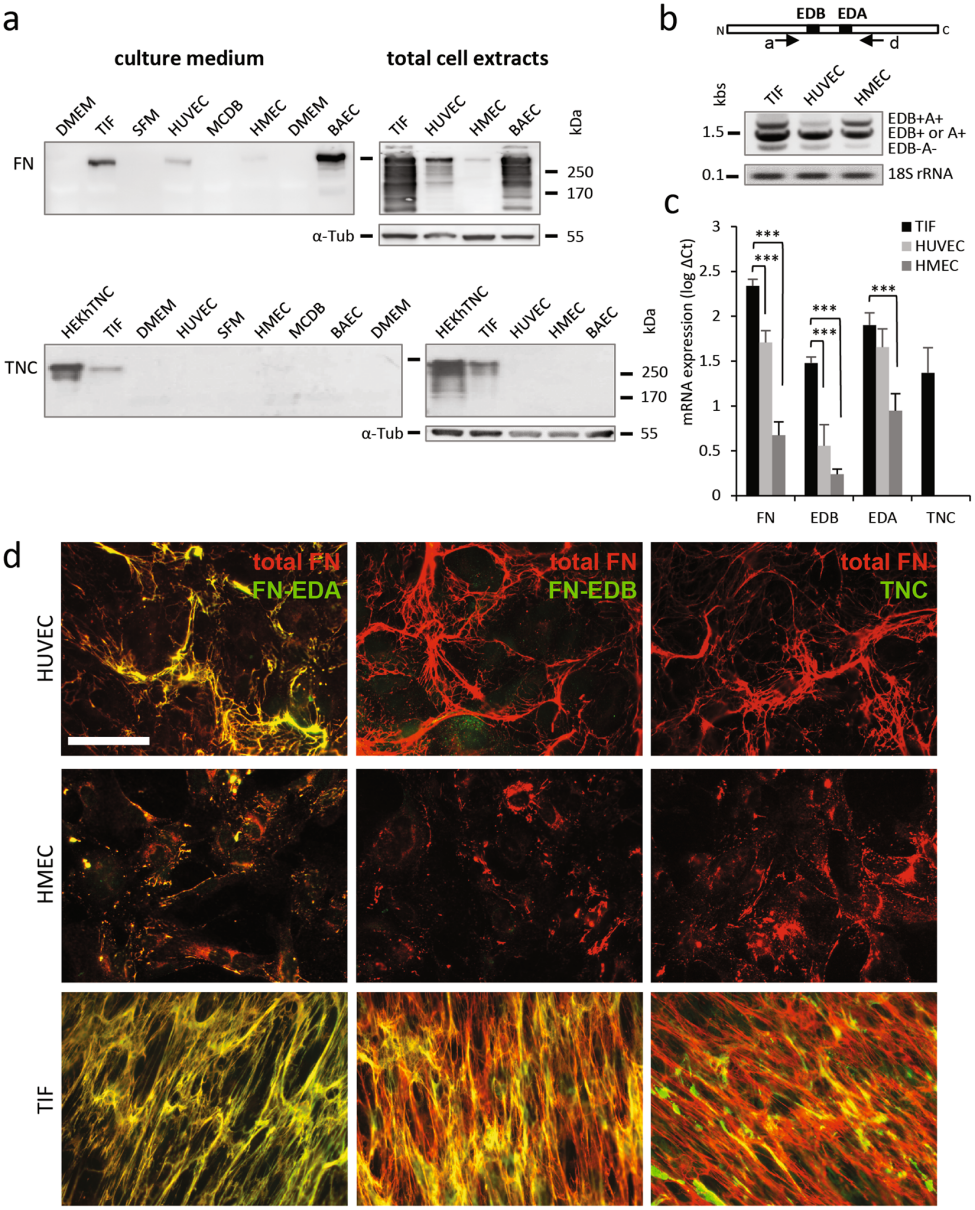
FN and TNC expression in different endothelial cell models. (**a**) Western analysis (cropped blots) of FN (top) and TNC (bottom) expression in indicated cells. Culture medium (25 µl), left; total cell extracts of equal protein concentration (40 µg), right. Equivalent amounts of FN-depleted serum-containing (10%) culture medium were deposited as control. α-tubulin was used as loading control for total cell extracts. Full-length blots are shown in Fig. S5. (**b**) Detection of cFN isoform transcripts (containing EDB+/EDA+, EDB+/EDA−, EDB−/EDA+, EDB−/EDA−, as schematically presented on the top) by RT-PCR in human endothelial cells and TIFs. 18S rRNA was used as internal control/housekeeping gene. Cropped blots are shown in (**a**) and (**b**). (**c**) QPCR analysis of FN, FN-EDB, FN-EDA and TNC mRNA expression in endothelial cells, normalized with GAPDH and presented as log ΔCt (±S.D., n = 5). (**d**) Double immunofluorescence staining (wide-field fluorescence) of total FN (red) and, in green, FN-EDA (left); FN-EDB (middle) or TNC (right) in confluent endothelial cells and TIFs. Cell density was evaluated after Hoechst 33342 staining (30–50 cells in each field). Bar = 100.

To study the functional interplay of FN and TNC at the cellular level, we first examined their expression in cultured endothelial cells of different origins (Fig. 2). The amount of soluble (secreted) and cell-associated protein was determined by Western analysis of conditioned culture medium and total cell extracts, respectively. As shown in Fig. 2a (top), different levels of FN were expressed by the endothelial cells examined (BAEC > HUVEC > HMEC). In each case, the majority of the expressed protein was present in cell lysates. We were unable to detect TNC expression by endothelial cells, under conditions in which it was detected in both conditioned medium and lysates from telomerase-immortalized fibroblasts (TIF) and from HEK293 cells expressing the recombinant protein, used as control (Fig. 2a, bottom).

FN associated with angiogenic tumour vasculature is known to harbour both alternatively spliced Extra Domains EDB and/or EDA^25,28^, yet the relative expression of the different variants by endothelial cells has not been clearly established. To address this issue, we analysed the presence of one or both Extra Domains in FN mRNA from different endothelial cells by PCR and qPCR (Fig. 2b and c). Whereas transcripts containing both domains (or neither domain) were detected, mRNAs containing only one Extra Domain were most prevalent (Fig. 2b). FN-EDA was the most abundant variant expressed by all cells (Fig. 2c).

Immunostaining of confluent cells with variant-specific antibodies confirmed the differential expression of FN variants. As shown in Fig. 2d and Supplementary Fig. S2a, all of the FN fibres assembled by HUVECs, HMECs, HMVEC-d and TIFs contained FN-EDA. FN-EDB was barely detectable in the matrix assembled by endothelial cells, whereas it was abundant in the TIF-derived matrix, consistent with the higher expression of this variant by TIFs.

The fibrillar patterning of the matrix reflects the cytoskeletal organization of the cells that assemble it. Thus, matrix assembled by TIFs, highly contractile cells, was composed of uniformly thick parallel fibres, whereas the HUVECs assembled a heterogeneous array of curved and straight fibres of varying thickness and length (Fig. 2d). A similar pattern of expression and organization of cellular FN was observed in dermal microvascular endothelial cells (HMVEC-d) and BAECs (Supplementary Fig. S2a) and^27^. TNC was not detected in the matrix of any of the endothelial cells examined, in accordance with the lack of expression by these cells, but it partially co-localized with FN in the matrix of TIFs. Together, these *in vitro* findings are in line with the conclusion from tissue stainings that endothelial cells are most likely not the source of perivascular TNC *in vivo*. Therefore, we next addressed the effect of exogenous TNC on endothelial cells.

### Delayed adhesion to TNC affects endothelial cell motility, cell-cell and cell-matrix adhesions

**Figure 3:**
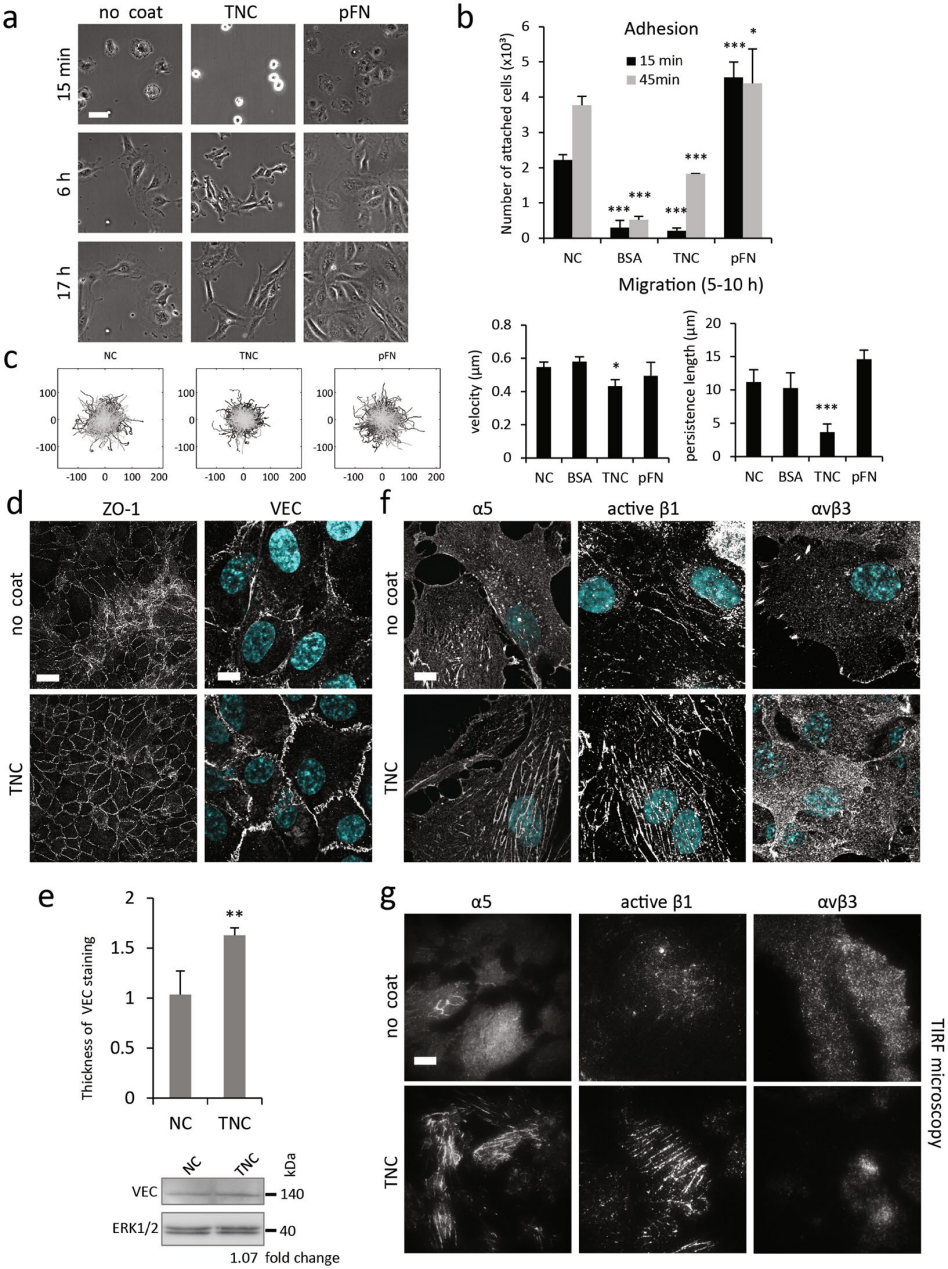
Effects of TNC on HUVEC adhesion and motility. (**a**) Phase contrast images of HUVECs seeded on a non-coated surface, 10 µg/ml of TNC or pFN (1 µg/ml) 15 min, 6 h and 17 h after plating. Bar = 50 µm. (**b**) Cell adhesion to a non-coated (NC) surface, or a surface coated with BSA, TNC (10 µg/ml) and pFN (5 µg/ml) was determined after 15 and 45 min of adhesion (±S.D., n = 3) (**c**) Sparsely plated cells were followed by time lapse video microscopy for 5 h (between 5 and 10 h after plating). Tracking of at least 100 cells per condition was performed using Imaris software and analysed in QuanTrack (left, tracings from origin). Histogram depicts the velocity (middle) and persistence length of cell movement on indicated substrates is shown on the right (±S.D., n = 5). (**d**) Imunnofluorescence staining of ZO-1 (wide-field fluorescence) and VE-cadherin (VEC) (confocal) was performed 48 h after plating of HUVECs on non-coated or TNC-coated coverslips. Bars = 50 µm (ZO-1) and 10 µm (VEC). (**e**) (top) Thickness of VEC staining in HUVECs plated on non-coated and TNC-coated coverslips was measured using the Integrated Morphometry Analysis module in MetaMorph software and results are plotted. (bottom) Cropped western blot of VEC in total HUVEC extracts. Full-length blots are shown in Fig. S6. (**f**) Cellular localization of integrins α5, active β1 and αvβ3 in HUVECs plated for 48 h on the indicated substrates was determined by immunofluorescence staining and confocal microscopy. Bar = 10 µm. (**g**) TIRF images of integrin (α5, active β1 and αvβ3) staining in HUVECs plated on the indicated substrates. Bar = 10 µm.

In accordance with the anti-adhesive properties of TNC, we observed a significant delay in adhesion of endothelial cells to TNC-coated plates, as compared to non-coated, or pFN-coated surfaces (Fig. 3a,b and Supplementary Fig. S2b). Under these conditions (complete medium containing 2% serum) most cells attached and spread on TNC, yet they retained distinct morphological features including stunted lamellipodial extensions, enhanced cell-cell cohesion and an elongated phenotype. Time-lapse video microscopy was performed to investigate adhesion dynamics, phenotypic features and motile behaviour of endothelial cells on TNC (Fig. 3a, Supplementary Fig. S2b and Movies 1–3). Quantrack analysis of cell tracking between 5 and 10 h after plating (illustrated in Fig. 3c and Supplementary Fig. S2c) revealed differences in cell migration on TNC. Notably, speed and persistence of migration was significantly reduced, as compared to a non-coated substrate (Fig. 3c, right and Supplementary Fig. S2c). Thus, cells moved more slowly in random directions.

At confluence, HUVEC cultured on immobilized TNC formed more organized cobblestone monolayers and displayed enhanced junctional staining of tight and adherens junction proteins, ZO-1 and VE-cadherin (VEC), respectively (Fig. 3d) and thicker VEC-based junctions, with no effect on expression of the protein (Fig. 3e).

We have previously reported that restricted endothelial cell motility together with increased intercellular cohesion was associated to signalling through an α5β1 integrin-FN axis, following α5β1 integrin-driven assembly of autocrine FN beneath cells^27^. To determine whether this could contribute to the observed TNC effect, we first analysed the expression and localization of integrins in endothelial cells plated on control (non-coated) or TNC-coated coverslips (Fig. 3f). Forty-eight hours after plating on uncoated glass coverslips, control cells displayed mostly punctate staining with some centrally-located α5β1 integrin adhesions. The number and length of the adhesions was markedly increased when cells were plated on TNC. Staining with anti-active β1 antibody suggested the presence of ligated α5β1 in the fibrillar adhesions. TIRF microscopy confirmed the basal localization of these adhesions as well as their increased abundance in cells exposed to TNC (Fig. 3g and Supplementary Fig. S3).

αvβ3 integrin was present in nascent adhesions and small focal complexes at the periphery of control cells, whereas staining of the integrin was largely diffuse in cells plated on TNC (Fig. 3f). Interestingly, in control cells the αvβ3 staining was readily detectable at the cell-substrate interface by TIRF microscopy, however the integrin signal was strongly reduced in TNC-exposed cells, suggesting that it lies beyond the evanescent field depth (e.g. in an intracellular recycling compartment or above a thick sub-endothelial matrix) (Fig. 3g). Together, these findings indicated that prolonged exposure to adsorbed TNC promotes the formation of α5β1 integrin-rich fibrillar adhesions and they prompted us to examine the effect of TNC on FN expression and assembly in endothelial cells.

### TNC enhances FN expression and impacts matrix patterning

**Figure 4:**
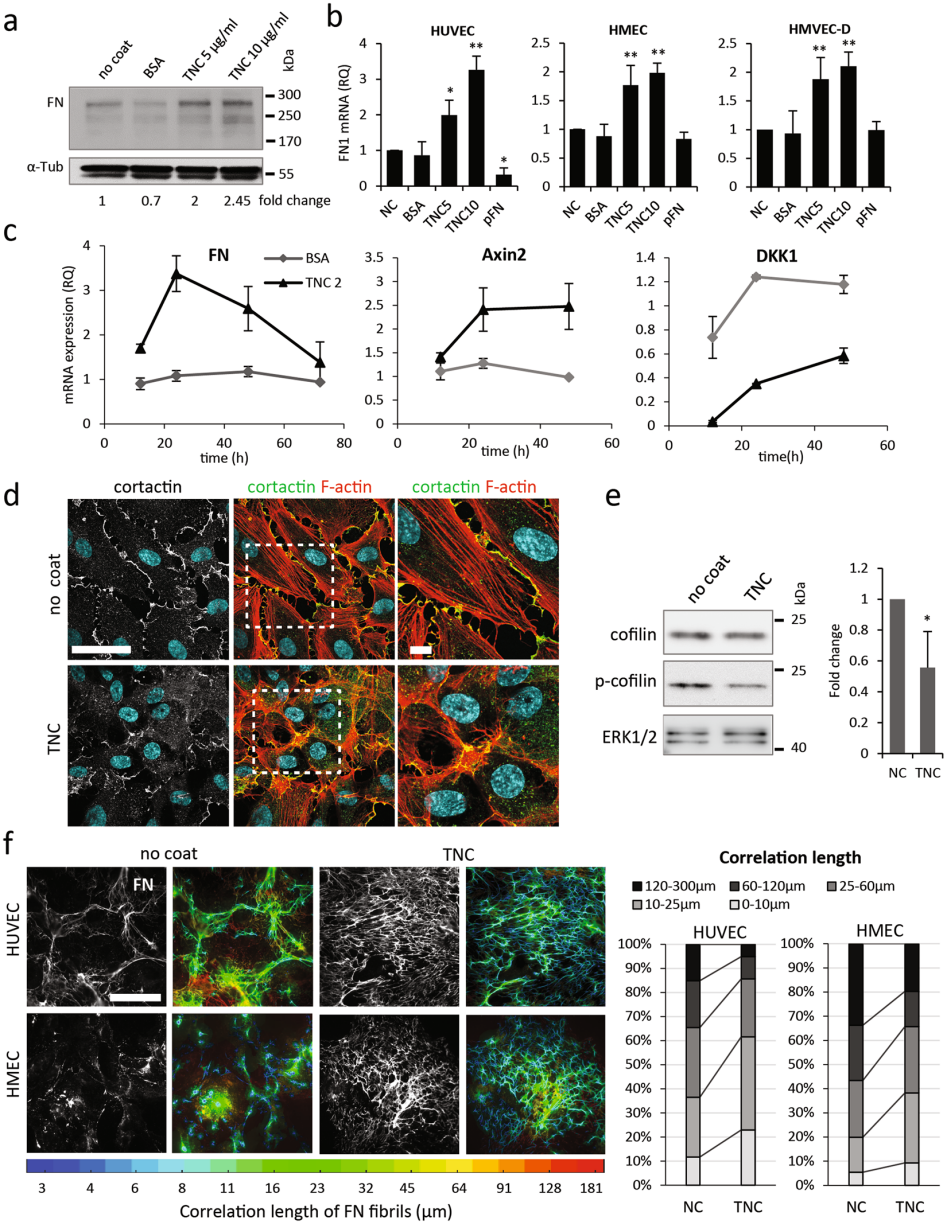
Effect of TNC on FN expression and assembly. (**a**) Representative western analysis of FN expression in total cell extracts of HUVECs cultured for 48 h on TNC-coated, BSA-pretreated or non-coated dishes. Equal amounts of protein (40 µg) were loaded in each lane and α-tubulin was used as loading control. Fold change over NC (normalized to α-tubulin) is shown below cropped blot. Full-length blots are shown in Fig. S7. (**b**) QPCR analysis of FN mRNA expression levels in different endothelial cells (left, HUVEC; HMEC, middle and HMVEC-d, right) cultured on indicated substrates in comparison to a non-coated (NC) surface (**c**). Time course (12–72 h) expression of FN (left), Axin2 (middle) and DKK1 mRNA (right) in HUVECs on TNC coating, non-coated or BSA-pretreated dish was monitored by QPCR. Fold change over NC (equal to 1, not shown on the graphs) was calculated using the ΔΔCt method (±S.D., n = 3). (**d**) Immunofluorescence staining of cortactin (white or green), filamentous actin (red) and nuclei (blue) of cells plated for 48 h on non-coated or TNC-coated coverslips. The zoomed area is indicated in white dotted square (right). Bar = 50 µm, and 10 µm in zoomed area. (**e**) Representative western blot analysis of cofilin phosphorylation at Ser 3 in total cell extracts of HUVECs cultured for 48 h on TNC-coated or non-coated dishes. Equal amounts of protein (40 µg) were loaded in each lane and ERK1/2 was used as loading control. Fold change over NC (normalized to ERK1/2) from 3 independent experiments is shown next to cropped blots. Full-length blots are shown in Fig. S7. (**f**) Immunofluorescence staining (wide-field images) of total FN in HUVECs (top) and HMECs (bottom) after 48 h of culture on non-coated or TNC-coated coverslips. FN fiber organization was analysed with homemade software coded in Matlab. Representative color-coded images of FN fibres in endothelial cells grown on indicated substrates are shown next to wide-field images. Color represents length of the fibres in µm (blue to red). At least 10 images were quantified for each condition and cumulative distribution of correlation length for HUVECs (upper right) and HMECs (bottom right) are plotted. Bar = 100 µm.

Assembly of the extracellular FN matrix takes place at fibrillar α5β1 integrin adhesions beneath endothelial cells. As previously established^27^, autocrine cFN expression plays a determinant role in this process. Accordingly, we observed that plating endothelial cells on TNC induced an increase in the expression of FN mRNA and protein in the different endothelial cells examined (Fig. 4a,b and Supplementary Fig. S4a). In HUVECs, the TNC-induced increase in FN mRNA expression was detected as early as 12 h after adhesion, it peaked at 24 h and returned to near basal levels after 72 h (Fig. 4c). Expression of both EDA- and EDB-containing variants was increased on a TNC substrate (Supplementary Fig. S4b), whereas this was not the case on BSA, another non-adhesive protein substratum. FN-EDB expression remained low in HUVECs and was only poorly detected in the sub-endothelial matrix (Supplementary Fig. S2a).

FN is an established canonical Wnt target gene^29^. TNC has been found to activate Wnt signalling in tumour cells and endothelial cells^30^. Therefore, we speculated that TNC may regulate FN expression in endothelial cells through Wnt signalling. In line with these findings, the expression of Axin-2 mRNA (another bona fide Wnt target gene^29^) was also increased following adhesion of HUVECs to TNC (Fig. 4c). Mechanistically, TNC-induced Wnt signalling has previously been shown to occur by downregulating expression of Dickkopf-1 (DKK1)^30^, a canonical Wnt signalling inhibitor. Indeed, TNC induced a rapid downregulation of DKK1 mRNA expression in HUVECs that preceded the induction of Wnt signalling and upregulation of FN expression (Fig. 4c). Together, these results suggest that TNC induces FN expression through Wnt singalling.

TNC inhibition of DKK1 expression was reported to occur through the blocking of actin stress fibre formation^30^. As shown in Fig. 4d, TNC induced a decrease in longitudinal actin stress fibres in cells plated on adsorbed TNC, as compared to cells on non-coated coverslips. The Rho/ROCK pathway is known to participate in the formation of actomyosin filament bundles and TNC has previously been shown to antagonize activation of the Rho/ROCK pathway by various agonists^31^. Accordingly, we observed a decrease in the phosphorylation of the ROCK substrate cofilin in endothelial cells plated on TNC, as compared to non-coated surfaces (Fig. 4e). Attenuation of cofilin Ser3 phosphorylation reflects an increase in the actin-severing activity of the protein. In addition to inhibiting stress fibre formation, TNC coating was found to negatively impact cortical actin polymerization. This can be seen by the redistribution of the cortical actin-binding protein cortactin from the cell periphery (in control cells) to the cytoplasm (in cells on TNC), as shown in Fig. 4d. Consequently, endothelial cells plated on TNC were less spread and displayed less cortactin-enriched edge-ruffles.

Both autocrine FN production and Rho-signalling have been shown to impact FN assembly. Therefore, we analysed the effect of increased FN expression and decreased ROCK activity on FN matrix deposition in endothelial cells plated on TNC (Fig. 4f, left and Supplementary Fig. S2a). Interestingly, we observed a dense array of thin highly-branched FN matrix beneath cells plated on TNC coats. This pattern of assembled fibres was reminiscent of the matrix deposited by RhoA-deficient endothelial cells, described in^32^, and consistent with our previous observations that α5β1 integrin translocation and FN fibril elongation can occur in low tension states resulting from RhoA depletion (decreased actomyosin filament bundles and contractile forces), similar to those encountered by newly-forming vessels in tissue. To analyse the FN fibre assemblies deposited by endothelial cells on TNC, we used a software coded in Matlab to generate color-coded images of fibre length and distribution. The results illustrated in Fig. 4f demonstrate that the fibres assembled by endothelial cells grown on TNC-coated surfaces are considerably shorter than those assembled by cells on non-coated coverslips. Hence, in the case of HUVECS on adsorbed TNC over 60% of the FN fibres measured <25 µm, versus less than 40% in absence of TNC. This tendency can be clearly visualized by the predominance of blue and green fibres in the color-coded images. Together, these findings demonstrate that exposure to TNC, through inhibitory effects on cytoskeletal remodelling, impacts not only the abundance of the FN matrix assembled by endothelial cells but also fibre patterning.

### TNC-conditioned FN matrix enhances capillary-like tube-forming ability of endothelial cells

**Figure 5:**
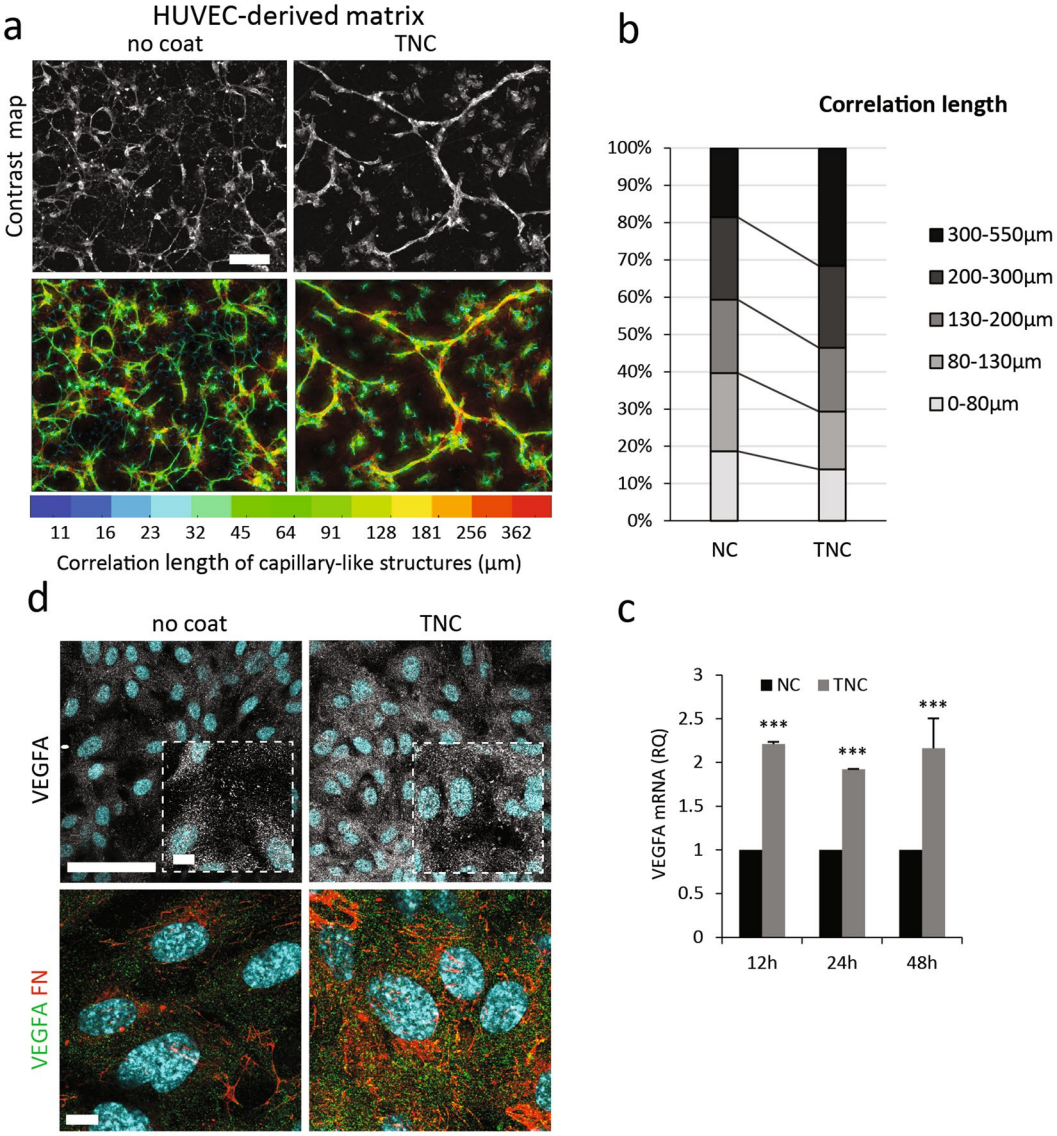
The effect of TNC pre-conditioned HUVEC-derived matrices on naive HUVECs. (**a**) A local contrast map of representative phase contrast images of naïve HUVECs seeded onto matrices prepared from HUVECs plated on non-coated (NC) or TNC-coated substrates (top) for 24 h. Color-coded correlation length (µm) is shown (below). (**b**) Distribution of correlation length is plotted. At least 15 images were quantified for each condition. (**c**) Time course of VEGFA mRNA expression in HUVECs plated for the indicated time on non-coated or TNC-pretreated dishes. Fold change over NC was calculated using the ΔΔCt method (±S.D., n = 3). (**d**) Immunofluorescence staining of VEGFA, FN and nuclei in HUVECs plated for 5 days on non-coated or TNC-coated coverslips. Dotted squares in upper panels depict zoomed areas. VEGFA could be detected in focal adhesion-like structures in areas of low cell density. Bar = 50 µm (upper panels), 10 µm (zoomed area and lower panels).

We next assessed the functional consequences of the dense highly-branched FN fibre network assembled by TNC-conditioned endothelial cells. To do so, we prepared decellularised matrices from HUVECs seeded on TNC-coated or non-coated dishes and observed the ability of naïve HUVECs to form capillary-like structures when plated on them. As shown in Fig. 5a and Supplementary Movies 4–5, matrix derived from HUVECs cultured on TNC increased capillary-like alignment of naïve HUVECs, as compared to matrix prepared from cells plated on non-coated plates. The cord-like structures formed after 24 h by endothelial cells grown on TNC-conditioned matrices were less branched (Fig. 5a) and longer than those assembled by cells on non-TNC-educated matrices. Thus only 18% of the cord-like arrangements were >300 µm long in absence of TNC (yellow/red structures in the color-coded images) versus more than 30% on TNC-conditioned matrices, as quantified in Fig. 5b. Modified capillary-like tube formation by HUVECs cultured on TNC-preconditioned matrices was accompanied by a sustained increase in VEGFA mRNA expression (Fig. 5c), as well as an increase in expression and accumulation of the protein in the FN-rich matrix (Fig. 5d).

### Autocrine FN impacts TNC-induced effects on cell adhesion, spreading and migration

**Figure 6:**
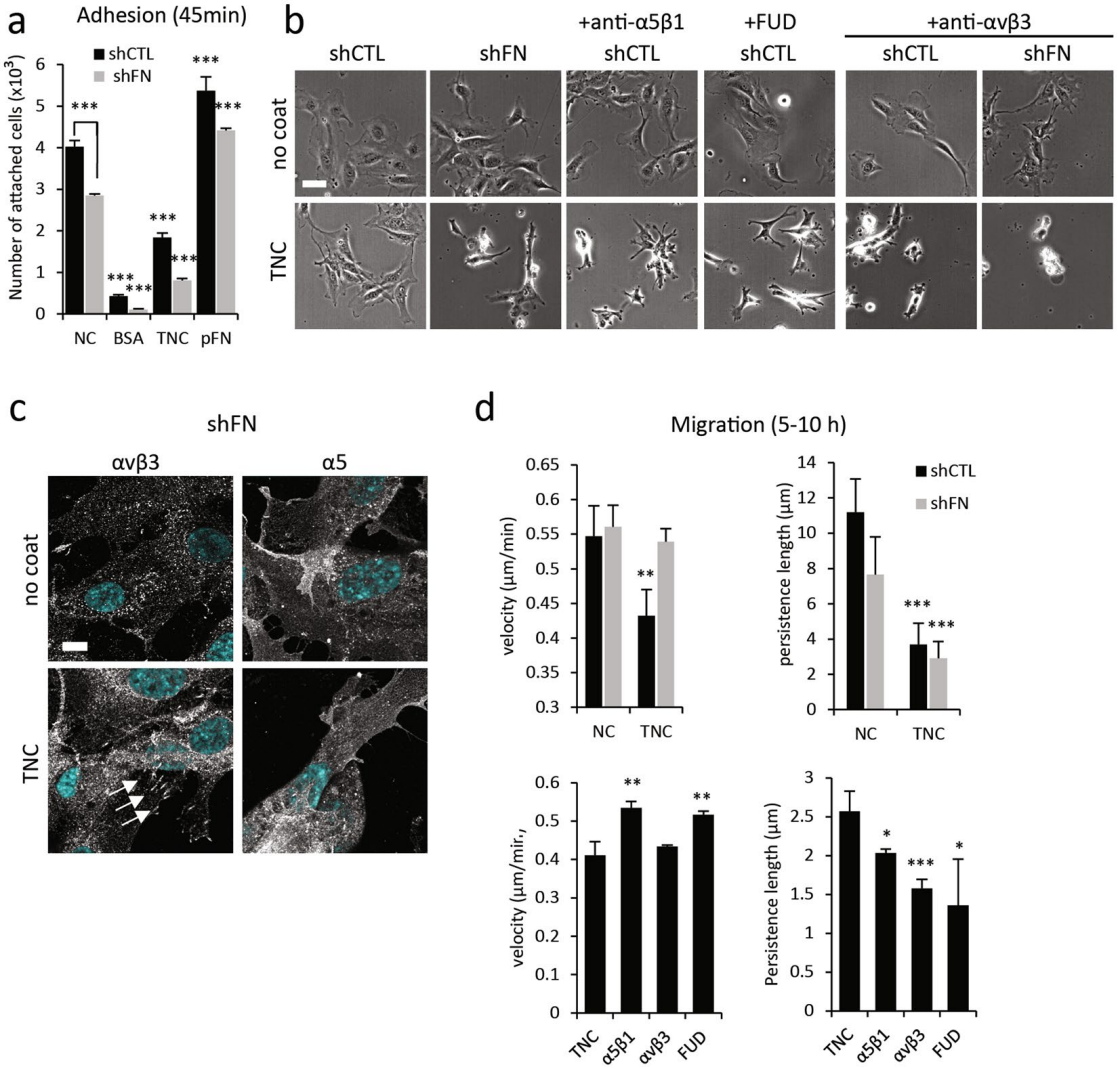
Effect of TNC on FN-deficient HUVECs. (**a**) Cell adhesion of FN-deficient (shFN) and control (shCTL) HUVECs to a non-coated (NC) surface, or a surface coated with BSA, TNC (10 µg/ml) or pFN (10 µg/ml) was determined 45 min after cell plating (±S.D., n = 3). (**b**) Phase contrast images of shCTL or shFN HUVECs pre-incubated with function blocking anti-integrin antibodies (anti-αvβ3 or α5β1) or with FUD peptide (300 nM) then cultured for 17 h on NC or on TNC. Bar = 50 µm. Additional images are shown in Supplementary Information **(**Figs S8–S10). (**c**) Cellular localization of integrins αvβ3 and α5 in FN-deficient HUVECs plated for 48 h on the indicated substrates was determined by immunofluorescence staining and confocal microscopy. Arrows indicate focal adhesions. Bar = 10 µm. (**d**) Sparsely plated cells were followed by time lapse video microscopy for 5 h (5–10h after plating). Tracking of at least 100 cells per condition was performed. Velocity and persistence length of shFN and shCTL cell movement was determined on non-coated or TNC-coated dishes (top) and on TNC-coated dishes (bottom), after pre-incubation with blocking anti-integrin antibodies (anti-αvβ3 and α5β1) or with FUD peptide (±S.D., n = 3).

To determine whether the observed effects of TNC on endothelial cells were dependent on FN expression and assembly, we prepared FN-deficient HUVECs by shRNA mediated knockdown. The efficiency of FN knockdown was controlled by quantitative real-time PCR (QPCR) (45%) and Western analysis (nearly 100%) (Supplementary Fig. S4c and d). First, the adhesion of FN deficient cells to TNC was examined (Fig. 6a and Supplementary Fig. S4e). After 45 min, adhesion of FN-deficient cells to TNC was detected albeit adhesion was reduced by up to 50% as compared to control (shCTL) cells (Fig. 6a). These results suggest that autocrine FN significantly participates in endothelial cell adhesion to TNC. Adhesion of shFN cells to non-coated or pFN-coated plastic also decreased, indicating that autocrine FN-mediated adhesion is not specific to TNC. Next, we performed time-lapse video microscopy to monitor the role of autocrine FN expression and assembly on endothelial cell migration. Phase contrast images in Fig. 6b show that cell spreading after an overnight incubation on TNC is clearly compromised in FN knockdown cells, and in control cells in which FN assembly is inhibited by preincubating cells with α5β1 integrin blocking antibodies or with the Functional Upstream Domain (FUD) of *Streptococcus pyogenes* adhesin F1^33^. Blocking αvβ3, a TNC-binding integrin (see^13^), only slightly reduced the spreading of control and FN-deficient cells on non-coated surfaces. However, it drastically perturbed the adhesion of shCTL cells on TNC and completely abrogated the adhesion and survival of shFN cells. Similarly, no cell adhesion to TNC was observed in presence of αvβ3- and α5β1-blocking antibodies (not shown). These results indicate that αvβ3 may be determinant for FN-independent endothelial cell adhesion to adsorbed TNC. Indeed, αvβ3 integrin was detected in adhesive structures (focal contacts and focal adhesions) in FN-depleted cells plated on TNC whereas α5β1, mainly unligated in these cells, was diffuse and absent from adhesive structures (Fig. 6c).

Cell migration analyses, shown in Fig. 6d, indicate that FN expression is responsible for the reduced velocity of endothelial cell migration on TNC. Hence, FN knockdown restored migration speed on TNC to values observed in cells on non-coated dishes. Similarly, the presence of agents that inhibit FN assembly (α5β1 integrin blocking antibodies or FUD) increased migration velocity. These results corroborate our previous findings in BAECs that FN expression/assembly restricts cell motility^27^. In contrast, the TNC-induced decrease in persistence of migration appears to be independent of FN expression and assembly. Blocking αvβ3 integrin in HUVECs plated on TNC had no effect on migration speed but further decreased the persistence length of cell trajectories, due to severe adhesion defects.

## Discussion

**Figure 7:**
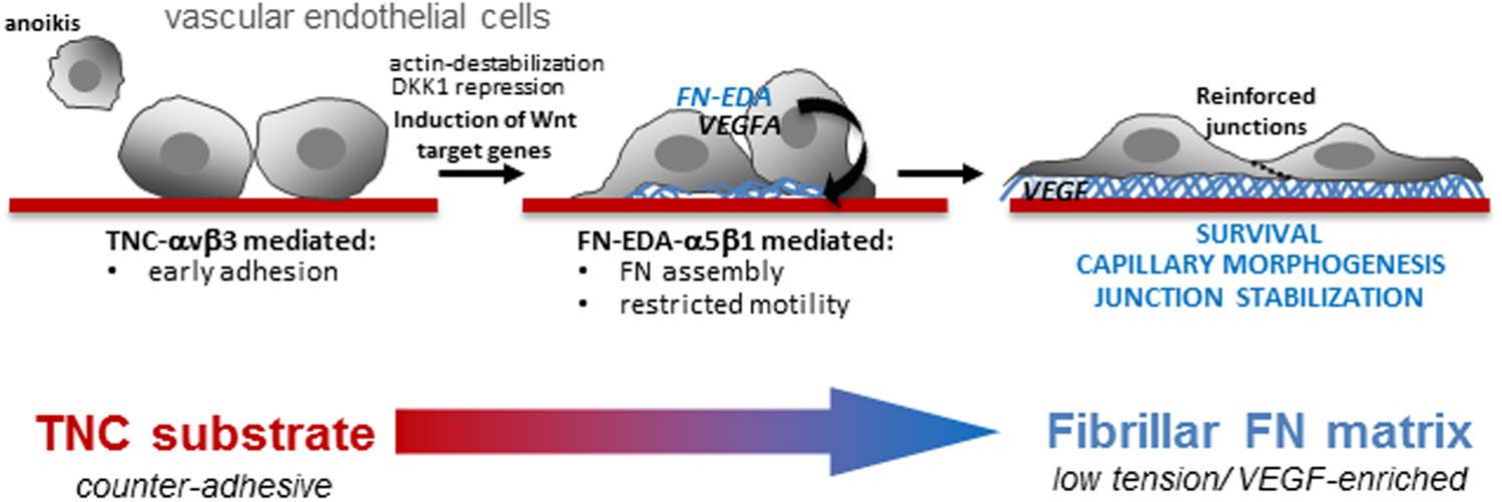
Schematic of TNC-regulated pro-angiogenic signalling in endothelial cells. TNC inhibits endothelial cell adhesion, spreading and stress fibre formation (F-actin polymerization). This effect can lead to anoikis, or be counterbalanced by upregulation of Wnt target genes including FN and VEGFA. Autocrine FN (EDA) drives α5β1-dependent FN fibrillogenesis, promotes junction stabilization and impacts capillary morphogenesis.

In this study we set out to decipher the functional interplay of TNC and FN in tumour angiogenesis at the level of endothelial cells. We found that TNC is not expressed in endothelial cells and exposure of cells to the immobilized protein stimulates expression and subendothelial assembly of autocrine FN. The newly assembled FN matrix promotes adhesion, protects cells from anoikis and reinforces junction stability (schematized in Fig. 7). Further, our results suggest that TNC-regulated expression of cellular FN contributes to the temporally ordered changes in matrix composition that impact vascular stability and maturation during development and dynamic remodelling of tumour-associated vessels.

Endothelial cells are highly dependent on cell adhesion for survival and direct exposure of endothelial cells to TNC has been shown to induce anoikis^11^. Here we describe a mechanism by which endothelial cells overcome anoikis that involves TNC-induced activation of Wnt signalling, previously observed in tumour and endothelial cells^30^. We extend this observation by identifying FN as an important TNC-induced Wnt response gene in endothelial cells. In the vasculature, canonical Wnt signalling has been shown to control endothelial cell proliferation, survival and vasculature stability through junction assembly and pericyte recruitment (reviewed in^34^). It is tempting to speculate that autocrine FN plays a decisive role in these Wnt/β-catenin-regulated events. We previously showed that FN expression in cultured endothelial cells is tightly coupled to basal assembly of the protein and to reinforcement of cell-cell junctions^27^. Hence, when FN expression is blocked cells are no longer able to assemble a subendothelial matrix (even in presence of added soluble pFN) and they lose their ability to establish stable cell-cell junctions and apico-basal polarity. Consequently, FN-depleted endothelial cells adopt a mesenchymal-like morphology. Interestingly, cell-cell junctions can be restored in FN-deficient endothelial cells by plating them on a FN-coated surface (*i*.*e*. basal anchoring of α5β1 integrin) thus bypassing the need for FN expression/assembly^27^. Our collective observations from past studies and the present report, support the notion that the induction of FN expression, involving canonical Wnt signaling, is a key mechanism by which TNC enhances FN fibrillogenesis and cell-cell adhesion in endothelial cells. In support of this idea, FN was shown to be a major target of Wnt signalling involved in the pulmonary vasculature during early lung development and DKK1 was a negative upstream regulator^35^, which is consistent with our observation of reduced expression of DKK1 by TNC in endothelial cells.

TNC has been associated with matrix deposition in the vasculature, where it plays a protective role in stress adaptation. Hence, deletion of the TNC gene renders mice susceptible to acute aortic dissection development upon aortic stress due, in part, to insufficient induction of ECM proteins^36^. It remains to be seen whether FN may be an important stress response gene in this context. In a mouse carotid ligation model, FN was reported to exert effects against haemorrhage of the vessel wall in response to low and disturbed arterial flow^37^.

Previous studies have addressed the inhibitory effects of TNC on cell attachment to a FN substratum^12,31,38^. In the latter reports, TNC was found to abrogate cell spreading on adsorbed FN by blocking the integrin α5β1/syndecan-4 complex. In the present study, we examined the direct adhesion-disrupting effects of TNC on endothelial cells, in absence of adsorbed FN. Importantly we show here that exogenous FN, in the case of HUVECs, shuts down autocrine production of the protein in a feed-back inhibitory loop. This may explain not only why TNC-regulated FN induction was not observed previously, but also why TNC-induced FN mRNA expression returns to basal levels following the assembly of a FN matrix in HUVECs.

Significant differences in FN fibril arrangements were observed beneath endothelial cells exposed, or not, to TNC and an increase in the tubulogenesis-promoting activity of the TNC-educated matrix. It is possible that direct molecular interactions between TNC and FN could impact autocrine FN assembly, via syndecan-4-dependent or independent mechanisms, once the FN is deposited. In any case, the observed differences in matrix patterning in cells plated on TNC are likely to stem from TNC-induced cytoskeletal modifications linked to Rho protein inhibition^12,39^. We have previously shown that RhoA-depleted endothelial cells, devoid of longitudinal stress fibres, maintain intact intercellular junctions and circumferential F-actin^32^. Interestingly, RhoA-depleted cells deposit a FN meshwork that resembles the FN matrix assembled by endothelial cells plated on TNC. These results, together with the known inhibitory effect of TNC on RhoA, provide mechanistic insights into the mechanism(s) by which TNC influences FN fibrillogenesis. *In vivo*, this could translate into modifications in biologically relevant properties of the matrix, including changes in physical and mechanical traits that could serve to tune growth factor/chemokine sequestration and presentation in TNC-rich perivascular environments. Here, we observed increased expression of VEGFA in HUVECs in response to TNC that coincided with enhanced capillary-like tube forming activity of naive HUVECs plated on the TNC-educated matrix.

In sum, our findings suggest a novel mechanism whereby contact with a counter-adhesive matrix protein can leverage a gene expression program in endothelial cells to modulate angiogenic blood vessel remodelling.

## Materials and Methods

### Materials and reagents

Plasma FN was from BD Biosciences (Bedford, MA, USA). Recombinant human TNC (large TNC variant containing all FNIII type domains except AD1 and AD2 domains) was obtained as described^12^. Alexa Fluor 647-conjugated phalloidin and Hoechst 33342 were purchased from Invitrogen (Eugene, Oregon, USA). DNase I was purchased from Roche (Penzberg, Germany) and ascorbic acid from Sigma-Aldrich (Missouri, USA). The plasmid encoding the FUD peptide from adhesin F1 of *Streptococcus pyogenes* that blocks FN matrix assembly was provided by Jane Sottile (University of Rochester). Recombinant FUD was produced and purified as described^33^.

### Antibodies

The following antibodies were used: polyclonal anti-FN, monoclonal anti-αvβ3 integrin (clone LM609) and blocking monoclonal anti-α5β1 integrin (clone JBS5) from Millipore (Billerica, MA); polyclonal VEGFA antibody from Abcam (Cambridge, MA); monoclonal anti-FN (clone 10) and monoclonal anti-ZO-1 from BD Biosciences (Le Pont de Claix, France); monoclonal anti-FN (clone F1, used for IHC) from Abgent (San Diego, CA, USA); anti-EDA (IST-9) and anti-EDB (C6) from Sirius biotech (Genoa, Italy); monoclonal anti-tenascin-C (clone BC24) from Sigma Aldrich (Lyon, France); monoclonal anti-CD29/activated-β1 integrin (clone 9EG7) from BD Pharmingen™ (San Diego, CA), monoclonal anti-α-tubulin (B-5-1-2) from Invitrogen, anti-ERK1/2 (C-16) and anti-cortactin (H-191) from Santa Cruz Biotechnology (Santa Cruz, CA), anti-VE-cadherin from Bender MedSystems (Tebu-bio SA, Le Perray en Yvelines, France), anti-cortactin (clone AF11) monoclonal antibodies from Upstate (Chemicon International, Ltd., Hampshire, UK), monocloal anti-CD31 (PECAM-1, clone JC70A) from Dako France S.A.S. (Les Ulis, France), polyclonal anti-cofilin and anti-phospho-cofilin (Ser3) from Cell Signalling (Danvers, MA). Secondary antibodies coupled to horseradish peroxidase were from Jackson Immunoresearch Labs (West Grove, PA). Fluorescently-labeled (Alexa Fluor 488, 564 and 647-conjugated) secondary antibodies were purchased from Invitrogen. References of commercial primary antibodies and dilutions used in experiments are indicated in Supplementary Table 1.

### Immunostaining of tissue sections

Tissue samples embedded in paraffin blocks were obtained from the Biological Resource Centre of the Pathology Department, Centre Antoine Lacassagne, Nice. Informed written consent was obtained from all the patients in accordance with institutional guidelines and the experimental protocol, that complied with ethical and safety practices for research involving human tissues, was approved by the CPP Sud-Méditerranée V ethics committee (National Authorisation Number: AC-2014-2237). Immuno-staining was performed as described^40^. For fluorescence, slides were labelled with appropriate secondary antibodies (Invitrogen) followed by nuclear staining with DRAQ5 (Thermo Scientific) and then mounted in ProLong Gold antifade reagent (Invitrogen). Immunohistochemically stained sections were digitalized using a Hamamatsu NanoZoomer 2.0-HT Digital slide scanner (40X mode). Images were visualized and captured using NDP.view2:U12388-01 software. Fluorescently-stained sections were analysed and images acquired with a 10x/0.45 or 40x/1.1 W objective on a Zeiss NLO780 confocal system.

### Cells and culture conditions

Human umbilical vein endothelial cells (HUVECs) were prepared from fresh human umbilical veins as described^41^, and maintained in human endothelial SFM (Invitrogen, Cergy Pontoise, France) supplemented with 10% foetal calf serum (FCS), epidermal growth factor (EGF, 10 ng/mL; Invitrogen), bFGF (10 ng/ml, prepared in the protein purification facility of our institute), and heparin (10 ng/ml; Sigma, Saint Quentin Fallavier, France). HUVECs were used up to the 5th passage for all experiments. The human microvascular endothelial cell line CDC.HMEC-1 (HMEC), provided by F. J. Candal (Center for Disease Control and Prevention, Atlanta, GA) was maintained in MCDB131 (Invitrogen) supplemented with 12.5% FCS, glutamine (10 mM), EGF (10 ng/ml), bFGF (10 ng/ml), heparin (10 ng/ml), and hydrocortisone (1 g/ml; Sigma). HMEC cultures were used between passages 15 and 22. Primary human dermal microvascular endothelial cells (HMVEC-d) (Lonza, Amboise, France) were cultured according to the manufacturer’s recommendations in EGM™−2MV BulletKit™. Bovine aortic endothelial cells (BAECs) have been described in (Vouret-Craviari *et al*., 2004). Telomerase-immortilised fibroblasts (TIFs) were provided by J. Norman (Beatson Institute, Glasgow, UK) and cultured in DMEM (Invitrogen, Cergy Pontoise, France) supplemented with 20% FCS and 20 mM Hepes. HEK293 cells expressing recombinant TNC have been described elsewhere^42^. For experiments, cells were maintained in culture medium supplemented with FN-depleted fetal calf serum obtained using gelatin sepharose-4B (GE Healthcare, Uppsula, Sweden) columns. Cells were routinely tested, with negative results, for mycoplasmal contamination by PCR (Mycoplasma Plus, Stragegene, La Jolla, CA).

### Coating and cell-derived matrix preparation

Culture plates or glass coverslips were coated with 10 µg/ml plasma FN in PBS, 5 or 10 µg/ml TNC in PBS 0.01% Tween and 10 µg/ml RAA in PBS for 1 h at 37 °C, PBS-washed and then blocked with 0.5% heat denatured fatty-acid-free BSA in PBS for 45 min at 37 °C. Plates were air-dried under sterile conditions in the culture hood for 10 min. Substrate coatings were controlled by immunofluorescence staining with anti-TNC or anti-FN antibodies. Cell-derived matrices produced by HUVECs were prepared as described^43^.

### Lentiviral vector construction and transduction

The pLB2CPGm lentiviral vector harbouring FN- or luciferase-targeting (control) shRNA sequences has been described (Serres *et al*., 2014). Lentivirus production and cell transduction were performed as described^44^.

### PCR and quantitative real-time PCR (QPCR)

Total RNA was extracted from cells cultured in 3.5 or 6 cm dishes using NucleoSpin RNA XS kit (Marcherey-Nagel Eurl, Hoerdt, France) or TRIzol reagent (Life technologies, Carlsbad, CA, USA) according to the manufacturer’s protocol, and subsequently 0.5 µg–1 µg RNA was used for reverse transcription with High Capacity cDNA Reverse Transcription Kit (Applied Biosystems, Foster City, USA). PCR reactions were performed with primers specific to human FN, flanking the region upstream of EDB (oligo a) and downstream of EDA (oligo d), human 18S rRNA was used as housekeeping gene. QPCR reactions were performed using TaqMan Gene Expression assays (Applied Biosystems) containing fluorescent probes and human gene-specific primers for GAPDH (Hs99999905_m1), FN1 (Hs01565277_m1), FN-EDA (AJPAC8E PN4441114 and FN-EDB (AJQJBEM), TNC (Hs01115665_m1), VEGFA (Hs00900055_m1) as recommended by the manufacturer. All reactions were performed in triplicates, using the StepOnePlus real time PCR System and TaqMan Fast Advanced Master Mix (Applied Biosystems). Another set of PCR reactions was carried out using gene-specific primers for GAPDH (Glyceraldehyde 3-phosphate dehydrogenase), DKK1 and Axin2 with Fast SYBR Green Master Mix (Applied Biosystems). GAPDH was used as reference gene in all QPCR reactions. Relative quantification (RQ) of transcript levels was done using the ΔΔCt method and data are shown as fold change over control (2^−ΔΔCt^). Primer sequences are as follows: GAPDH, sense 5′-AGCGAGATCCCTCCAAAATC-3′, anti-sense 5′- GGCAGAGATGATGACCCTTT-3′; DKK1, sense 5′- GACCATTGACAACTACCAGCCG-3′, anti-sense 5′- TACTCATCAGTGCCGCACTCCT-3′; axin2, sense 5′- CCACACCCTTCTCCAATCC-3′, anti-sense 5′- TGCCAGTTTCTTTGGCTCTT-3′; FN- EDB, sense (oligo a) 5′-CCTGGAGTACAATGTCAGTG-3′, FN-EDA, anti-sense (oligo d), 5′-GGAGCAAGGTTGATTTCTTT-3′, 18S rRNA, sense 5′-TCGGAACTGAGGCCATGATT-3′, anti-sense 5′- CCTCCGACTTTCGTTCTTGATT-3′.

### Western blot analysis

Conditioned medium was harvested from cultures, or cells were lysed in 3x Laemmli buffer, 24 h or 48 h after plating. Proteins were separated by gel electrophoresis before transfer onto Immobilion P membranes (Millipore, Bedford, MA, USA) according to standard procedures. Immune complexes on membranes were detected by enhanced chemiluminescence (GE Healthcare Amersham, Fisher Scientific) using a Fusion FX7 analysis camera (Vilber Lourmat, Marne-la-Vallée, France) or directly on films. ERK1/2 or α-tubulin detection was used as loading control for cellular proteins. Western blots were quantified using Fusion FX7 software. Uncropped scans of representative blots are shown in Supplementary Figures 5–7.

### Cell adhesion

For cell adhesion assays triplicate wells of 48-well plates were coated with the indicated protein or peptide then blocked with 0.5% BSA in PBS. Cells were detached with trypsin-EDTA, re-suspended in medium with 2% serum and plated (5 × 10^3^ cells per well) for 15 or 45 min. Following removal of non-adherent cells, adhesion was quantified on crystal violet-stained cells using ImageJ 1.49m (Wayne Rasband, NIH, USA) software.

### Video microscopy and cell migration

Phase contrast imaging and video microscopy were performed using a 10X/0.25 air objective on a Zeiss Axiovert 200M microscope equipped with a sCMOS NEO camera (Andor, Belfast, UK). Image acquisition at 5 min intervals was performed using the MetaMorph Imaging System (Universal Imaging Corp., Downingtown, PA). Migration of cells was monitored by automatic tracking of fluorescently labeled non-dividing cells using Imaris 8.1 software (Bitplane AG, Zurich, Switzerland) with a tracking module. For analysis, we developed ‘Quantrack’, a Graphical-User-Interface standalone written in MatLab (The MathWorks) that is available upon request. Motility of at least 100 cells was analysed in each field for every condition, in the indicated time frame (between 5–10 h or 10–16 h) after cell plating. Three independent experiments were performed for each condition and data are presented as mean ± SEM.

### Fluorescence microscopy and image analysis

For immunofluorescence analyses, cells were fixed in 3% paraformaldehyde/3% sucrose and permeabilised with 0.2% Triton X-100. After staining, the coverslips were mounted in ProLong® Gold antifade reagent (Invitrogen) or directly in PBS (for TIRF microscopy). Wide-field fluorescence was observed through 40X/1.3 oil objectives on a Zeiss Axiovert 200M microscope equipped with a CoolSnap HQ CCD. Image acquisition was performed using the MetaMorph Imaging System. Confocal imaging was performed on a Zeiss NLO780 confocal system using a 63X/1.4 objective. Total Internal Reflection Fluorescence (TIRF) microscopy was performed on our prototype system including 100x/1.49O Nikon objective, Ixon3 EMCCD (Andor, Belfast, Northern Ireland) and a laser bench with 491 nm (Oxxius, Lannion, France) and 561 nm (Cobolt, Solna, Sweden).

Images were analysed using MetaMorph Imaging System and Volocity (PerkinElmer, Waltham, MA) software. Quantification of VEC staining thickness was performed with the Integrated Morphometry Analysis module in MetaMorph software.

### FN fibre and tube formation analysis

We developed a graphical user interface (GUI) program (coded in Matlab) to quantify filamentous behaviour of images. This program convolves the image with a set of kernels, each with a linear pattern. This pattern varying in length $L$ and orientation $\theta$, yields the 4D matrix ${Corr}(x,y,L,\theta )$. For each pixel $(x,y)$, the program extracts both the map of maximum correlation length ${L}_{{\max }}(x,y)$ and a mask (combining the correlation score and fluorescence intensity) $M(x,y)$. We color-coded in HSV (hue saturation value) where hue is ${L}_{{\max }}(x,y)$, saturation is 1, and the value is $M(x,y)$. Fluorescence images were analysed directly. For phase contrast images, a pretreatment of the image was required to create a local contrast map for analysis.

### Statistical Analyses

Statistical analysis was performed using unpaired *t*–tests. Statistically significant data are indicated by *(p < 0.05), **(p < 0.01) or ***(p < 0.001).

### Data availability statement

Data, materials, and protocols described herein will be made available upon request.

## Electronic supplementary material

Movie 1

Movie 2

Movie 3

Movie 4

Movie 5

Supplementary information
