# Supplementary material for: Counterbalancing anti-adhesive effects of Tenascin-C through fibronectin expression in endothelial cells
Source: Sci Rep. 2017 Oct 6;7:12762. doi: 10.1038/s41598-017-13008-9 (PMC5630602; doi:10.1038/s41598-017-13008-9)
Supplement: Supplementary file 6 — Supplementary information [file 41598_2017_13008_MOESM6_ESM.pdf]

Supplementary Information

"Counterbalancing anti-adhesive effects of Tenascin-C through fibronectin expression in endothelial cells"

Agata Radwanska, Dominique Grall, Sébastien Schaub, Stéphanie Beghelli-de la Forest Divonne, Delphine Ciais, Samah Rekima, Tristan Rupp, Anne Sudaka, Gertraud Orend and Ellen Van Obberghen-Schilling

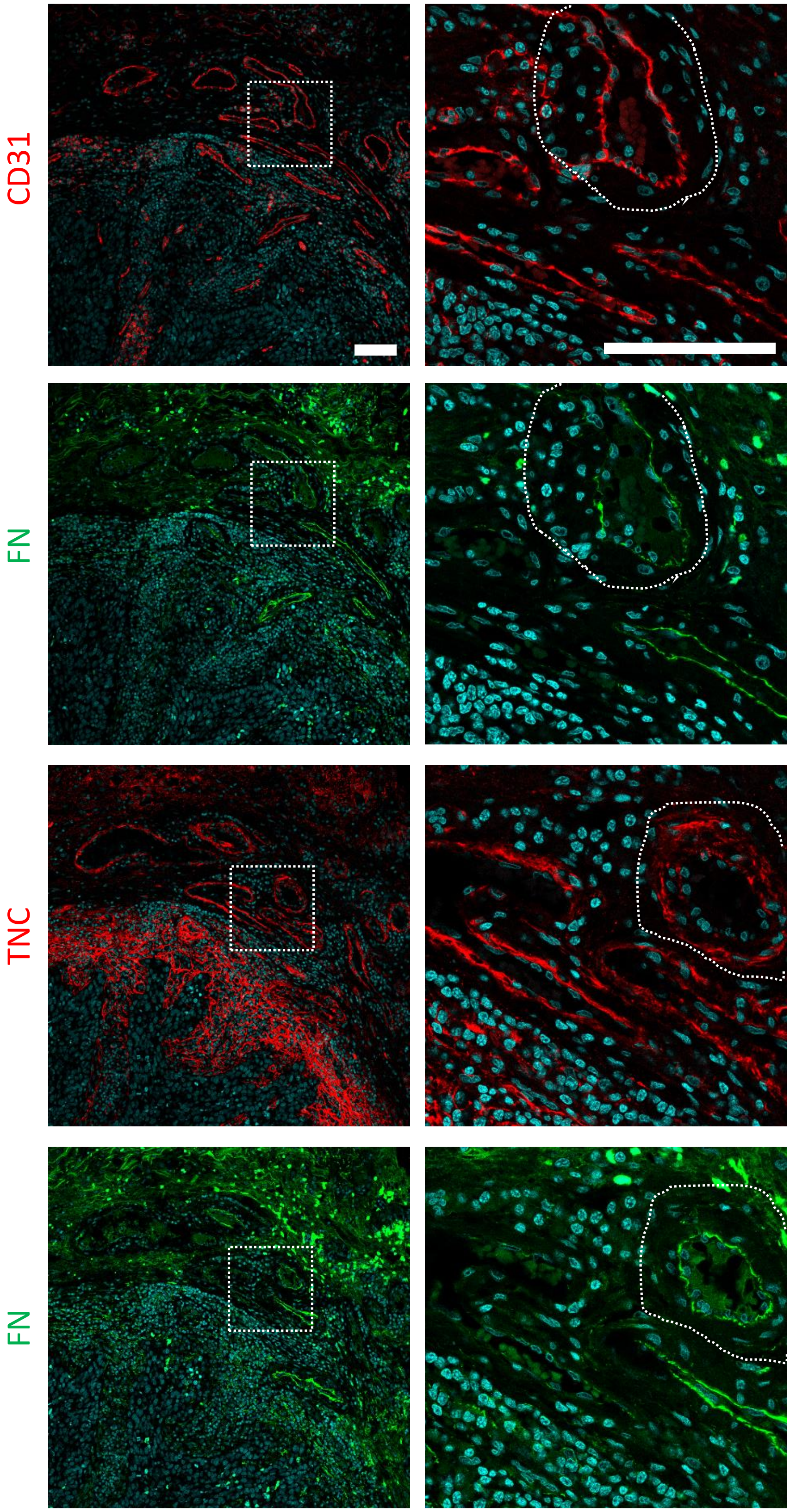
CD31
FN
TNC
FN

Figure S1 FN and TNC are expressed in angiogenic blood vessels of human tumours. Immunofluorescent staining, as indicated, of FN, CD31 and TNC on adjacent sections of the same human HNSCC tumour (separated green and red channels are shown). Nuclei are stained with DRAQ5 (blue). Dotted squares (left images) depict zoomed areas (right images). Corresponding areas of the same vessel are encircled. Bars=100 µm.

Supplementary Figure S2

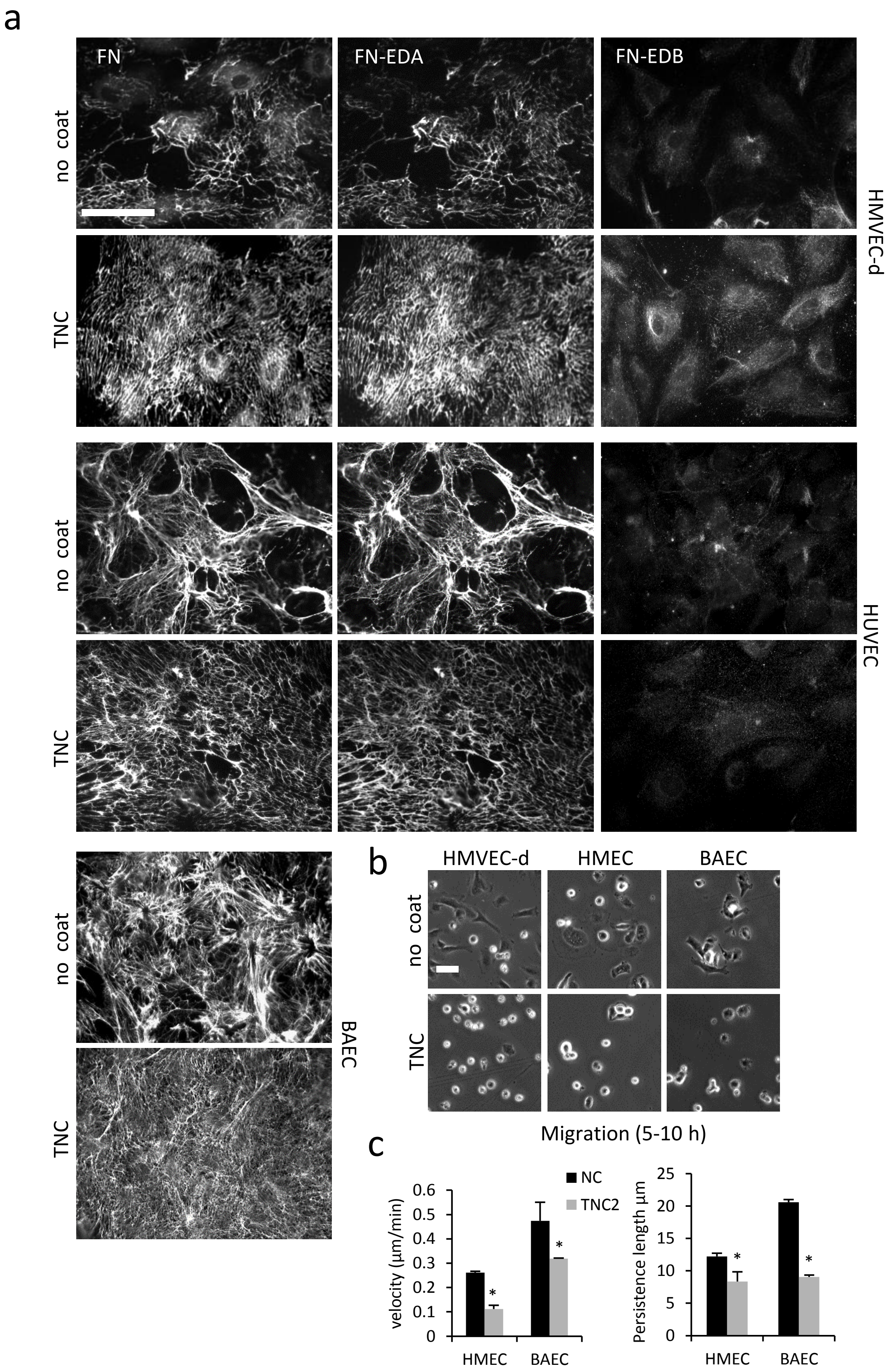

Figure S2 FN variant expression, cell adhesion and migration of endothelial cells on TNC.

(a) Immunofluorescence staining of total FN, FN-EDA and FN-EDA in confluent endothelial cells on non-coated or TNC-coated coverslips (wide-field fluorescence). Bar=100 µm. (b) Phase contrast images of endothelial cells seeded on non-coated (NC) or TNC-coated surfaces, 15 min after plating. Bar=50 µm. (c) Sparsely plated cells were followed by time lapse video microscopy for 5 h (between 5 and 10 h after plating). Tracking of at least 100 cells per condition was performed. Histogram depicts the velocity of cell movement on indicated substrates (top), persistence length is shown on the bottom. (±S.D., n=2).

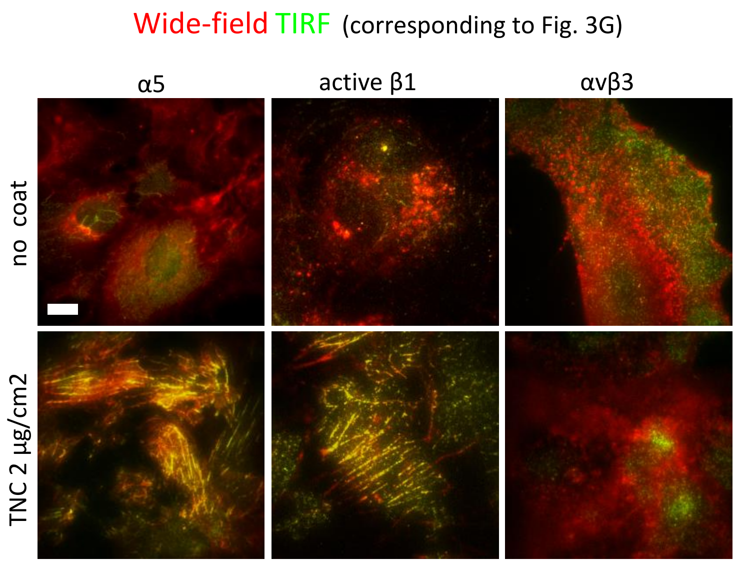

Figure S3 Cellular localization of integrins in HUVECs. Wide-field images (red) superimposed with corresponding TIRF images (green) of integrin (α5, active β1 and αvβ3) staining in HUVECs plated on the indicated substrates. Bar=10 µm.

Supplementary Figure S4

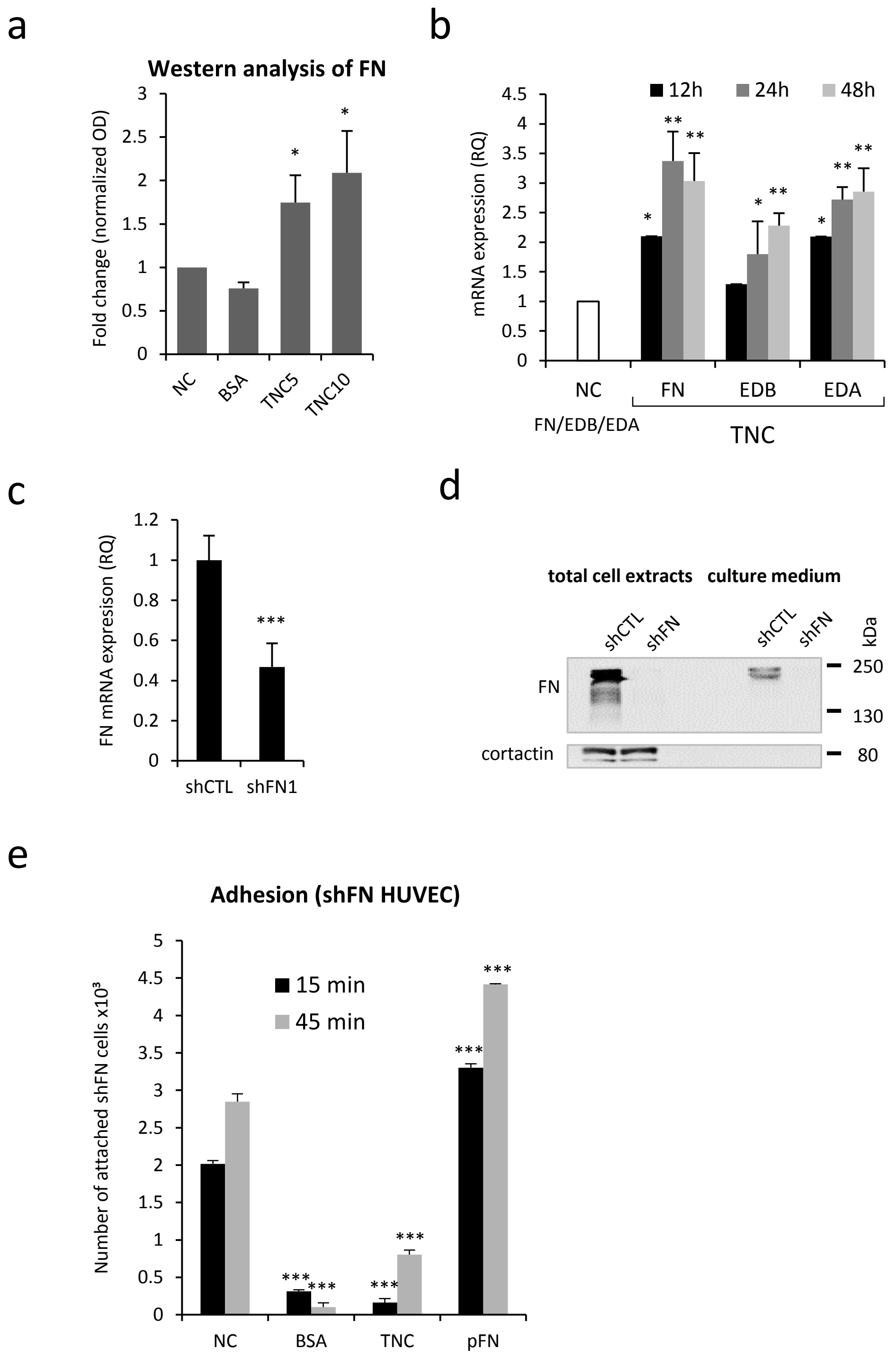

Figure S4 Characterization of FN variant induction and shRNA mediated knockdown of FN in HUVECs. (**a**) Quantification of western analysis of FN expression in lysates of HUVECs cultured on TNC- (5 and 10 µg/ml), BSA- or non-coated dishes for 48 h. Equal amounts of protein (60 µg) were loaded in each lane and a-tubulin was used as loading control. Fold change over NC (normalized to a-tubulin) is shown (n=3). (**b**) QPCR analysis of FN, FN-EDB, FN-EDA mRNA expression in HUVECs on TNC. Fold change (RQ) over NC (equal to 1, shown only once) was calculated using the ΔΔCt method (±S.D., n=3). (**c**) FN mRNA expression in shCTL and shFN HUVECs was controlled by QPCR. Fold change over shCTL was calculated using the ΔΔCt method (±S.D., n=3). (**d**) FN knockdown was verified by Western blotting in total cell lysates (40 µg) and conditioned medium (25 µl) collected from control (shCTL) and shFN cells. Cortactin was used as loading control. (**e**) Cell adhesion of FN-deficient (shFN) HUVECs to a non-coated surface, or a surface coated with BSA, TNC (5 and 10 µg/ml) or pFN (5 µg/ml) was determined 15 and 45 min after cell plating (±S.D., n=3).

Supplementary Figure 5

Representative western blots of FN and TNC in conditioned culture medium and total cell extracts (lysates) for Figure 2 a.

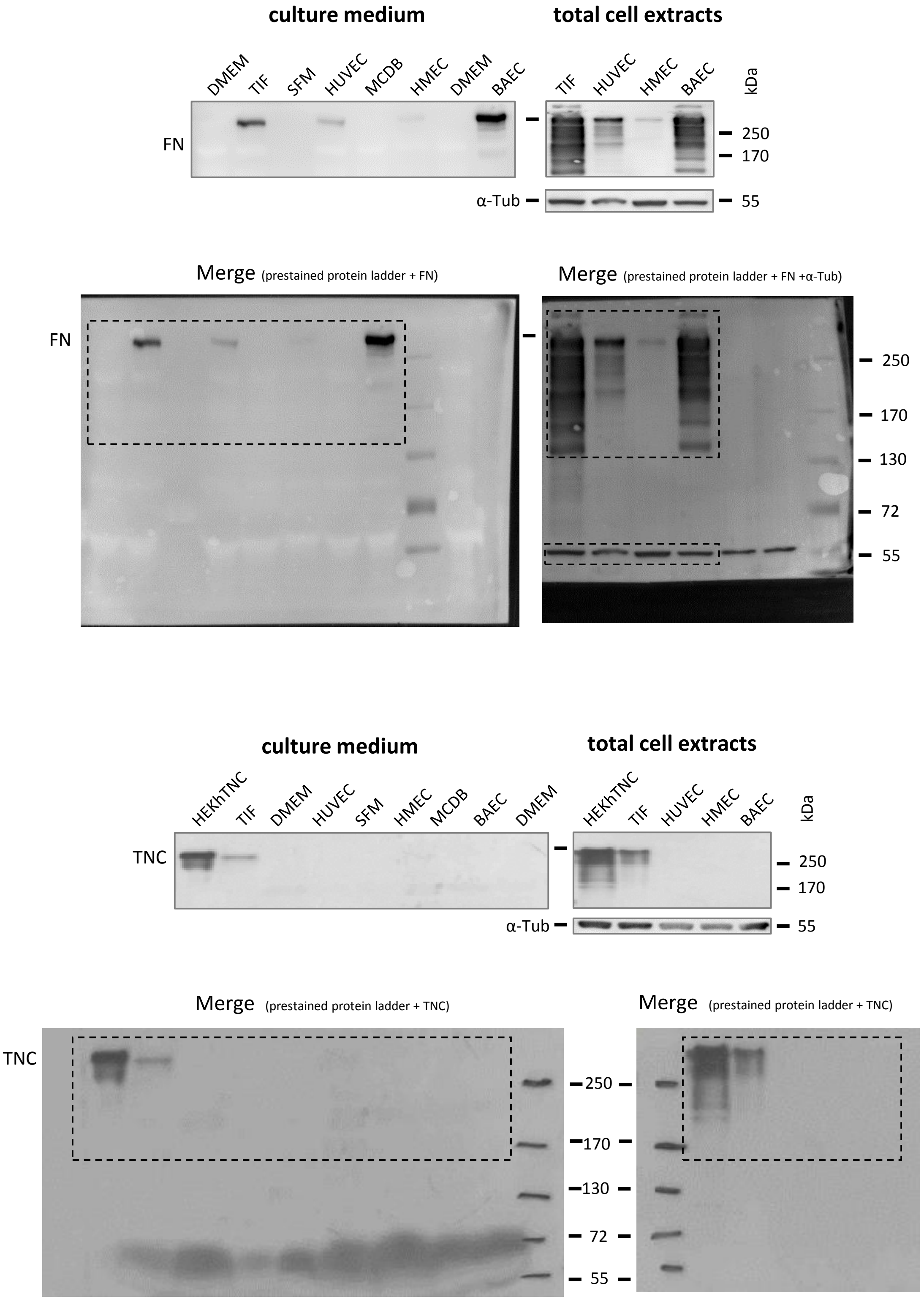

Supplementary Figure 6

Representative western blots of VEC total cell extracts (lysates) for Figure 3 e.

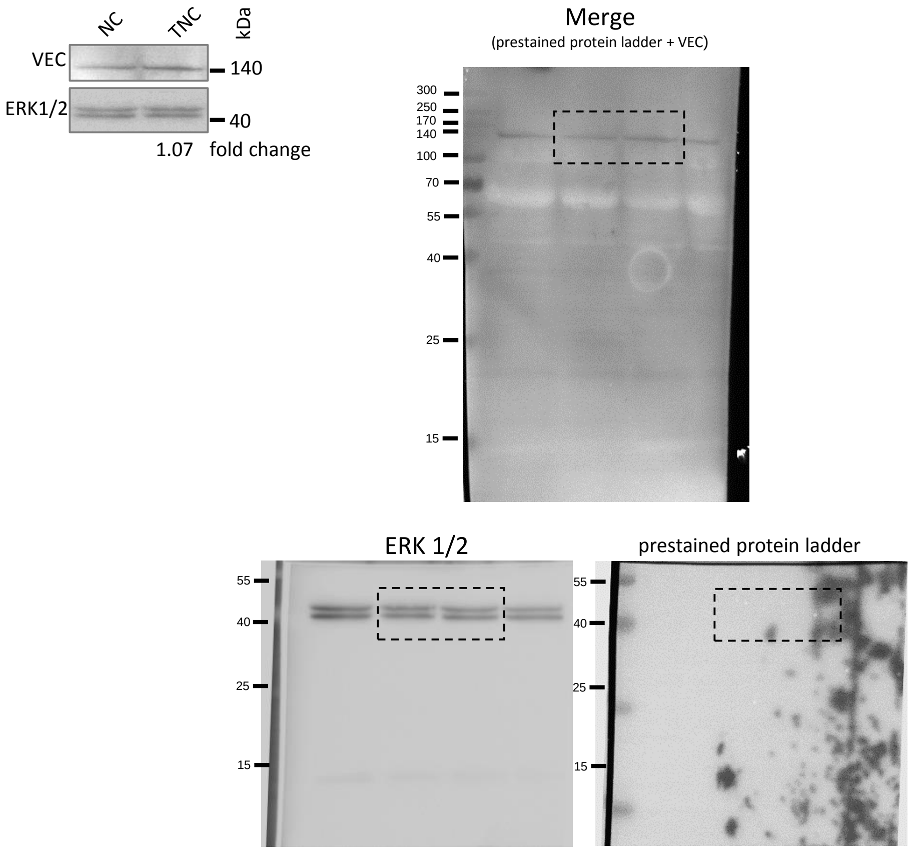

Supplementary Figure 7

Representative western blots of FN and α-Tub in total cell extracts (lysates) for Figure 4 a.

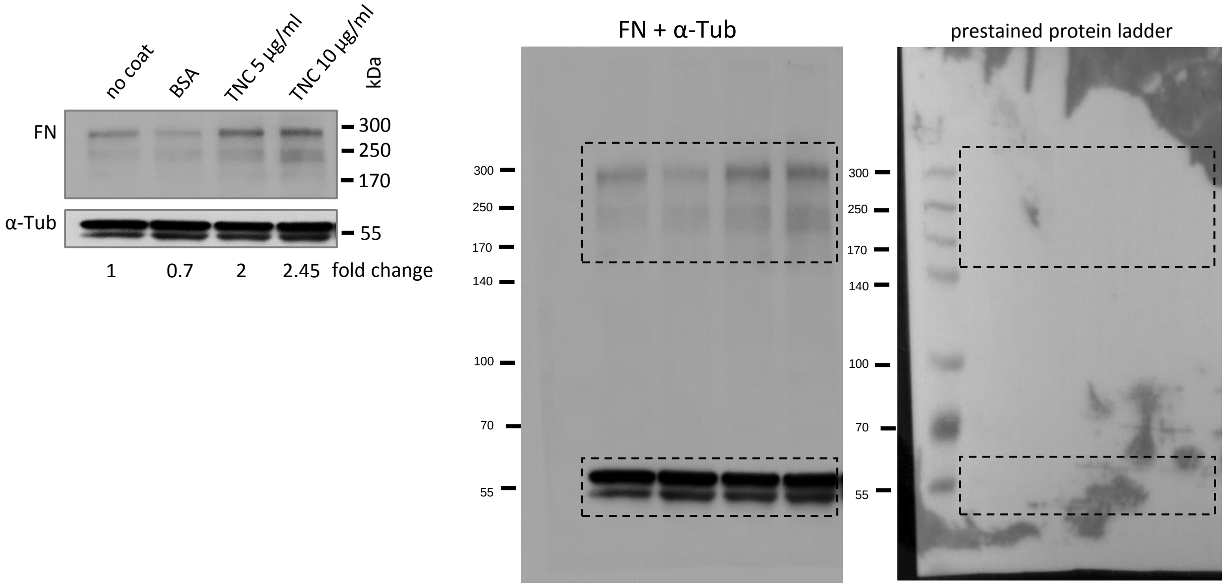

Representative western blots of cofilin and p-cofilin in total cell extracts (lysates) for Figure 4 e.

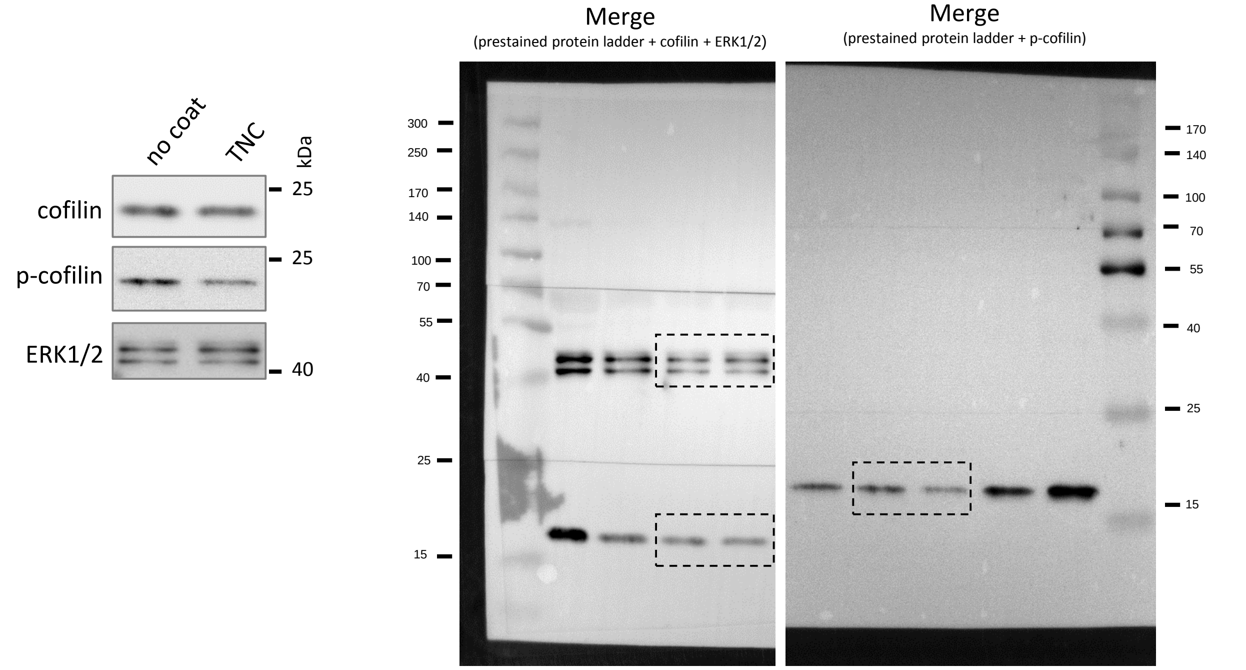

Supplementary Figure 8 Additional images for Figure 6b.

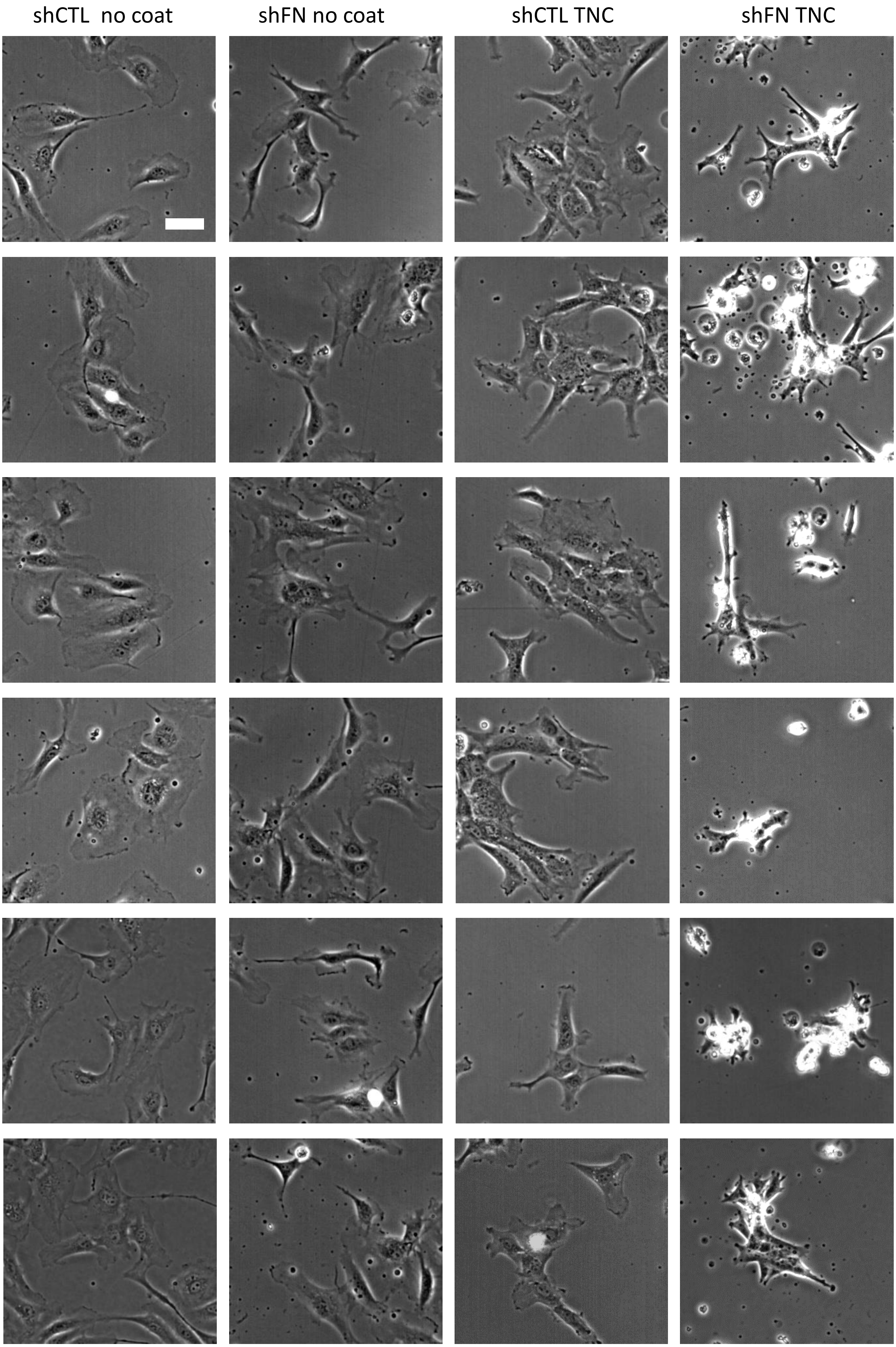

Supplementary Figure 9 Additional images for Figure 6b.

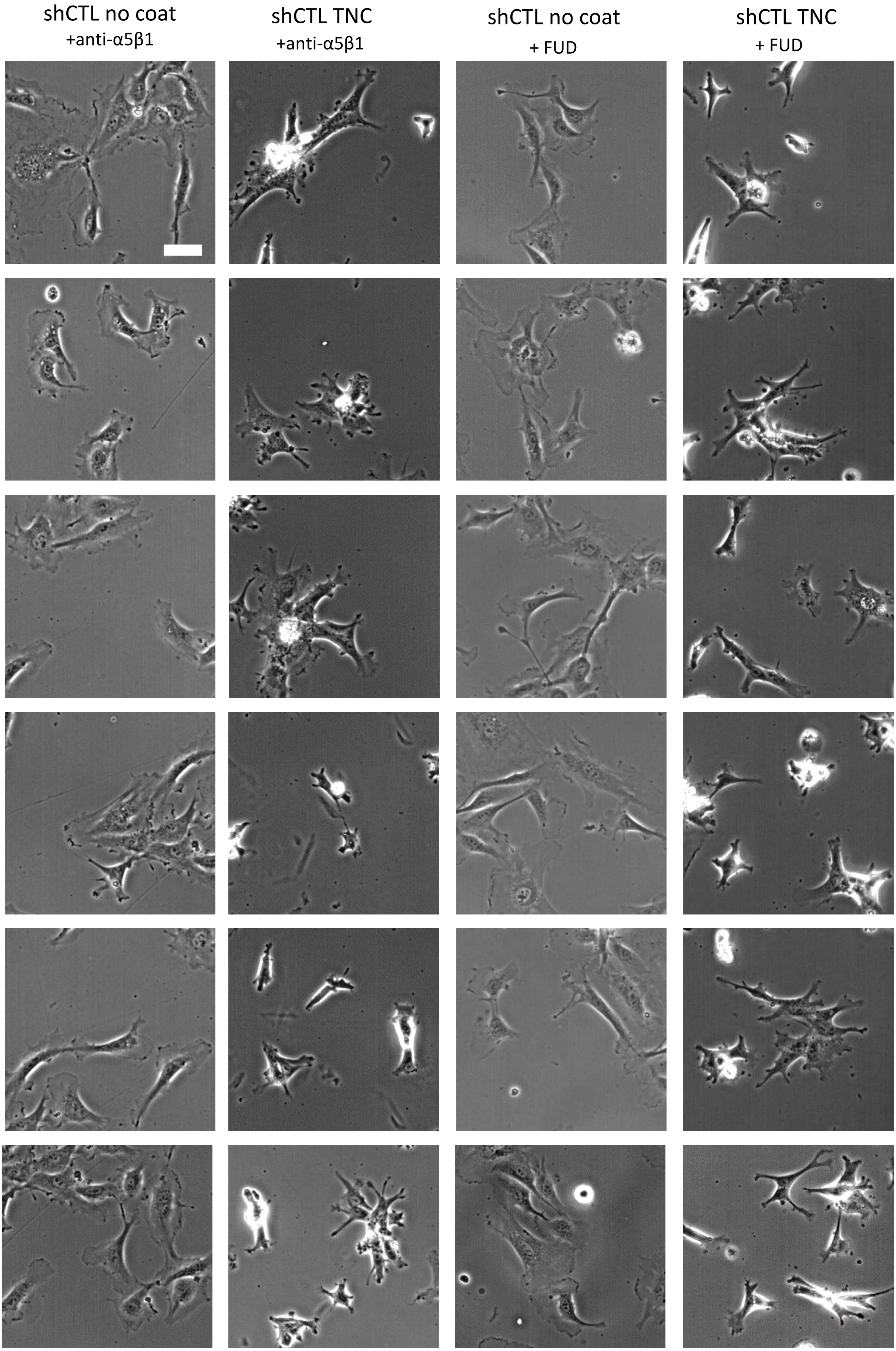

Supplementary Figure 10 Additional images for Figure 6b.

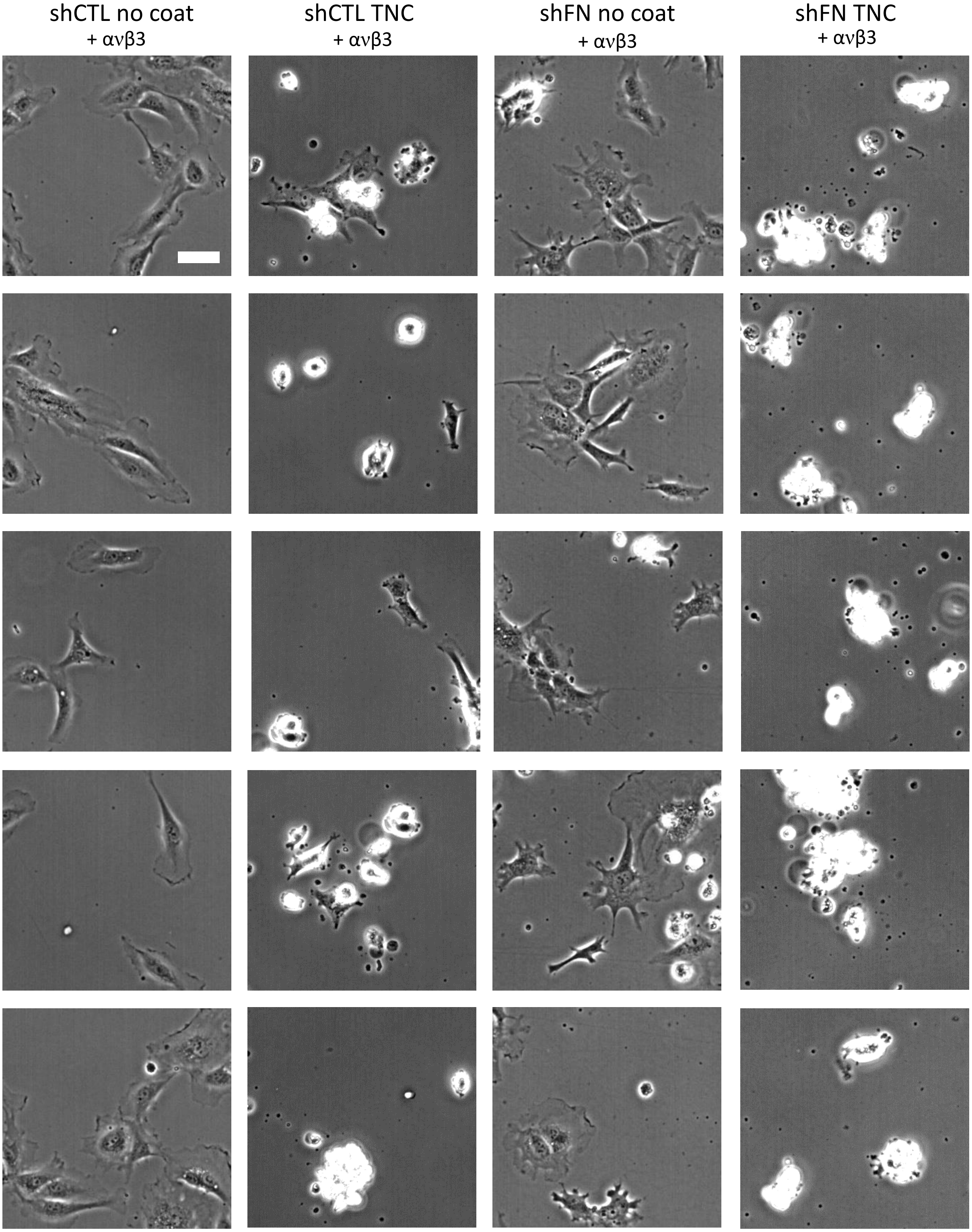

Supplementary Figures 8-10 Phase contrast images of shCTL or shFN HUVECs pre-incubated with function blocking anti-integrin antibodies (anti-αvβ3 or α5β1) or with FUD peptide then cultured for 17h on NC or on TNC. Bar=50 µm.

Supplementary Table 1

Primary antibodies used, suppliers and dilutions

Antibody	Clone (Reference)	Supplier (address)	Source	Application
Cofilin	(8503)	Cell Signalling Technology (Beverly, MA)	rabbit	WB 1:1000
phospho-Cofilin (Ser3)	(3311)	Cell Signalling Technology	rabbit	WB 1:1000
ZO1	(610966)	BD Biosciences (Le Pont de Claix, France)	mouse	IF 1 :250
FN	10(610077)	BD Biosciences	mouse	WB 1:4000<br>IF 1:500
TNC	BC24 (T2551)	Sigma-Aldrich (St. Louis MO)	mouse	WB 1:2000<br>IF 1:100<br>IHC 1:1000
VEGFA	(ab51745)	Abcam (Cambridge, MA)	rabbit	IF 1 :100
VE-cadherin	BMS158	Bender MedSystems Tebu-bio SA, France	rabbit	WB 1:1000<br>IF 1:500
FN-EDA	IST-9 (S-FN5)	Sirius biotech (Genoa, Italy)	mouse	WB 1:200<br>IF 1:100
FN-EDB	C6(S-FN12)	Sirius biotech	mouse	WB 1:200<br>IF 1:100
ERK1	C-16 (SC-93)	Santa Cruz Biotechnology (Santa Cruz, CA)	rabbit	WB 1:2000
cortactin H-191	(SC11-408)	Santa Cruz Biotechnology	rabbit	IF 1:100
FN	F1(AJ1297a)	Abgent(San Diego, CA)	mouse	IHC 1:100
FN	(AB1945)	Millipore (Billerica, MA)	rabbit	IF 1:400
αvβ3 integrin	LM609 (MAB1976)	Millipore	mouse	IF 1:100
α5 integrin	(AB1928)	Millipore	rabbit	IF 1:500
blocking α5β1 integrin	JBS5 (MAB1969)	Millipore	mouse	blocking 10µg/ml
Activated-β1 integrin	9EG7(553715)	BD Pharmingen™ (San Diego, CA)	mouse	IF 1:400
α-tubulin	B-5-1-2(32-2500)	Invitrogen (Eugene, Oregon, USA)	mouse	WB 1:2000
CD31	JC70A(M0823)	Dako(Les Ulis, France)	mouse	IHC 1:1000

Movie Legends

Movies 1-3. Sparsely plated HUVECs were followed by time lapse video microscopy for 24h, starting from cell seeding. Images were taken every 5 min. Movie 1, HUVECs on non-coated dish; movie 2, HUVECs on TNC coating; movie 3, HUVECs on pFN coating.

Movies 4-5. Naive HUVECs were plated on HUVEC-derived matrices and followed by time lapse video microscopy for 24 h starting from cell seeding. Images were taken every 5 min. Movie 4 HUVECs on control matrices; movie 5 HUVECs on TNC-enriched matrices (obtained from cells plated on TNC).
